# Establishment of an In Vitro DSS-Induced Epithelial Injury Model to Investigate Dose-Dependent Effects of IL-6 and Butyrate on Barrier Function

**DOI:** 10.3390/ijms27188028

**Published:** 2026-09-09

**Authors:** Srijal Kunwar, Saleh A. Naser

**Affiliations:** Burnett School of Biomedical Sciences, College of Medicine, University of Central Florida, Orlando, FL 32816, USA; srijal.kunwar@ucf.edu

**Keywords:** dextran sodium sulfate (DSS), inflammatory bowel disease (IBD), IL-6, Caco-2, cytokines, epithelial barrier, butyrate

## Abstract

Dextran sodium sulfate (DSS) is widely used in vivo to induce epithelial injury; however, the epithelial-specific responses to DSS and mechanisms associated with barrier restoration remain incompletely understood. Here, we establish an in vitro DSS-induced epithelial damage model using differentiated Caco-2 cell monolayers. Exposure to 2.5%(*w*/*v*) DSS for 24 h resulted in significant upregulation of the pore-forming protein *Claudin-2(CLDN2)* (2.677-fold) and elevated oxidative marker protein levels of NADPH oxidase 1 (NOX1), accompanied by increased reactive oxygen species (ROS) production confirmed via dihydroethidium (DHE) staining. Increased epithelial permeability was observed by transepithelial electrical resistance (TEER) analysis, without significant cytotoxicity. Surprisingly, ELISA analysis revealed significant downregulation of Interleukin-6 (IL-6) (0.364 ± 0.034 pg/mL) following DSS exposure. Treatment with 1 ng/mL recombinant IL-6 (rIL-6) improved epithelial barrier function during continued DSS exposure, whereas a higher dose of 5 ng/mL did not produce the same restorative response. Furthermore, treatment of DSS-damaged Caco-2 monolayer with 10 mM butyrate significantly improved epithelial barrier function. Collectively, this study establishes a reproducible DSS-induced Caco-2 epithelial injury model and demonstrates concentration-dependent effects of rIL-6 and barrier-restorative effects of butyrate, providing an epithelial-specific platform for investigating intestinal barrier dysfunction relevant to IBD.

## 1. Introduction

Inflammatory bowel disease (IBD), comprising ulcerative colitis (UC) and Crohn’s disease (CD), is a chronic relapsing inflammatory disorder of the gastrointestinal tract that affects millions of individuals worldwide and is on the rise, especially in newly industrialized nations [1]. While CD is characterized by transmural discontinuous inflammation and can affect any part of the gastrointestinal tract, UC is mainly restricted to the colonic mucosa. Both conditions have similar pathological characteristics, such as persistent intestinal inflammation, poor mucosal healing, and progressive epithelial damage, despite variations in disease distribution and histopathology. These characteristics ultimately raise the risk of colorectal cancer and significantly lower patients’ quality of life. While considerable advances have been made in understanding the immunopathogenesis of IBD, the mechanisms regulating epithelial injury and mucosal repair remain incompletely understood. Finding mechanisms that support barrier repair has become a major focus of IBD research since long-term disease remission is increasingly linked to restoration of epithelial barrier function rather than only suppression of inflammation.

The intestinal epithelial barrier serves as the first line of defense against the luminal environment by preserving a tightly controlled interface between host tissues and intestinal microorganisms. The barrier is made up of a single layer of intestinal epithelial cells that are covered in mucus that allows nutrients to be absorbed selectively, restricting the passage of bacteria, toxins, and other luminal antigens. Disruption of epithelial integrity results in increased intestinal permeability, commonly referred to as a “leaky gut,” that allows microbial products to penetrate the mucosa and initiate or perpetuate inflammatory responses. Persistent barrier failure worsens the symptoms of IBD, including inadequate nutrient absorption, dysregulated immune activation, and delayed tissue regeneration [2]. Intercellular junctional complexes, especially tight junctions, control paracellular permeability by maintaining the integrity of the epithelial barrier [3]. During inflammatory conditions, barrier integrity is lost, which leads to increased epithelial permeability. Claudin-2, one of the tight junction proteins, has drawn particular attention due to its role as a pore-forming protein that enhances paracellular ion and water permeability and is constantly elevated in intestinal tissue that is inflamed. On the other hand, these proteins are often employed as markers of epithelial barrier integrity in experimental settings because disruption or redistribution of ZO-1 from the epithelial junctional complex is linked to decreased transepithelial electrical resistance (TEER) and poor barrier function [4,5].

According to several reported studies, oxidative stress dysregulates the epithelial barrier during intestinal inflammation. Similarly, reactive oxygen species (ROS), which are involved in normal cellular signaling and host defense under physiological settings, can overpower endogenous antioxidant defenses during chronic inflammation and lead to damage to proteins, lipids, and nucleic acids that jeopardizes epithelial integrity [6,7]. Additionally, it has been demonstrated that oxidative stress modifies the expression and localization of tight junction proteins, increasing intestinal permeability and compromising epithelial healing by altering ZO-1 organization and encouraging Claudin-2 expression [5]. The NADPH oxidase (NOX) family, especially NOX1, which is abundantly expressed in intestinal epithelial cells and is crucial for redox signaling and host defense, is one of the main enzymatic sources of ROS in the intestinal epithelium [8]. During inflammation, NOX1 activation is dysregulated, which has been linked to excessive ROS generation, epithelial injury, and intestinal barrier failure, suggesting oxidative stress as a crucial mechanism connecting epithelial damage to disease progression, even though physiological NOX1 activity contributes to epithelial homeostasis. Identifying signaling molecules that control inflammatory responses and epithelial repair has received a lot of attention since oxidative stress plays a major role in epithelial damage. Among these, interleukin-6(IL-6) is one of the most well-researched cytokines in intestinal inflammation. Numerous immune and non-immune cells, such as macrophages, T cells, fibroblasts, endothelial cells, and intestinal epithelial cells, release IL-6, which activates the JAK/STAT pathway to produce a range of physiological consequences [9]. Since increased IL-6 expression and signaling are regularly seen in the inflamed intestinal mucosa and circulation of IBD patients, especially those with Crohn’s disease, IL-6 has historically been predominantly thought of as a pro-inflammatory cytokine [10]. Persistent IL-6 signaling increases the survival of pathogenic T cells, stimulates immune cell activation, and intensifies inflammatory pathways that lead to chronic intestinal inflammation [11].

Nevertheless, new research indicates that IL-6’s biological roles go beyond its well-known pro-inflammatory function. Additionally, IL-6 can support epithelial survival, proliferation, and mucosal regeneration after injury, depending on the cellular context and signaling route involved. While trans-signaling via the soluble IL-6 receptor has been linked to maintaining chronic inflammation and the advancement of disease, classical IL-6 signaling via the membrane-bound IL-6 receptor is often linked to tissue protection and epithelial healing [12,13]. These contradictory roles have made it challenging to target IL-6 therapeutically and suggest that the biological effects of IL-6 are significantly influenced by the cellular setting and stage of sickness. Therefore, it is essential to understand the dual role of IL-6 during epithelial damage and restoration of barrier function to develop therapy strategies that restrict pathological inflammation without compromising mucosal repair.

Short-chain fatty acids, which are microbial-derived metabolites, have received particular attention lately due to their anti-inflammatory, antioxidant, and barrier-protective roles, including butyrate. Butyrate has been shown to preserve tight junction organization, increasing transepithelial electrical resistance (TEER), and reduce intestinal permeability [14,15,16]. Furthermore, butyrate has also shown promising effects in reducing oxidative stress and inflammatory signaling, thereby creating a favorable environment for epithelial healing following injury [17,18]. Consistent with these findings, our previous work also demonstrated that butyrate restored epithelial barrier integrity and reduced NOX1 and Claudin-2 expression in an in vitro inflammatory model using conditioned media from *Mycobacterium avium* subsp. *paratuberculosis* (MAP)-infected macrophages [19]. Although this model differs from the DSS-induced epithelial injury model used in the present study, these findings provide complementary evidence for the effects of butyrate on epithelial oxidative stress and tight junction regulation. Collectively, these observations support further investigation of butyrate as a potential therapeutic strategy for promoting restoration of barrier function despite continued DSS exposure.

Dextran sodium sulfate (DSS) is one of the most widely used chemical agents to induce intestinal epithelial injury and barrier dysfunction, providing a useful experimental system for evaluating epithelial barrier-restorative responses. DSS can directly cause damage to the intestinal epithelial layer and make it leaky, thereby mimicking several pathological features of IBD, including oxidative stress, tight junction disruption, and increased epithelial permeability [20,21]. Although DSS-induced colitis has been extensively studied in animal models, epithelial injury in vivo is influenced by complex interactions among immune cells, stromal cells, and the intestinal microbiota, which makes it difficult to distinguish epithelial-specific mechanisms of injury and repair [22,23]. In contrast, differentiated Caco-2 monolayers provide a controlled in vitro system that mimics key structural and functional characteristics of the intestinal epithelium, allowing controlled investigation of epithelial injury and barrier-restorative responses. Previous studies have established that DSS can directly affect the function of the Caco-2 epithelial barrier and have used this system to model epithelial injury [24,25]. However, most studies have primarily characterized specific parameters, for instance, permeability and tight junction protein remodeling, without concurrently assessing oxidative stress pathways or their relationship with cytokine signaling. Existing Caco-2 models have also largely been used to test single-mechanism interventions, rather than to dissect the interplay between oxidative stress (NOX1), tight junction remodeling (claudin-2), and IL-6 signaling within a single, reproducible injury and barrier restorative framework [25,26]. Furthermore, reported IL-6 responses to DSS in Caco-2 models remain inconsistent. Some studies have reported a significant increase in IL-6 secretion after DSS exposure, while others report only a weak cytokine response [24,27,28]. In the present study, DSS exposure led to a reduction in IL-6 secretion, a finding that, to our knowledge, has not been previously reported in this model and underscores the need for a well-characterized, reproducible model that allows epithelial cytokine responses to be interpreted in conjunction with concurrent measurements of oxidative stress and tight junction integrity rather than in isolation. Therefore, the present study established and characterized a reproducible DSS-induced epithelial injury model using differentiated Caco-2 monolayers to investigate epithelial barrier dysfunction and restoration during continued DSS exposure. Using this model, we evaluated epithelial permeability, effects on oxidative stress, tight junction remodeling, and IL-6 secretion following DSS exposure and subsequently assessed the effects of rIL-6 and butyrate on epithelial barrier restoration during continued DSS exposure. By providing a controlled epithelial-specific injury model, this study offers a practical platform for investigating mechanisms of epithelial healing and evaluating therapeutic strategies for inflammatory bowel disease.

## 2. Results

### 2.1. DSS Treatment Induces Time- and Dose-Dependent Epithelial Barrier Dysfunction

**Figure 1:**
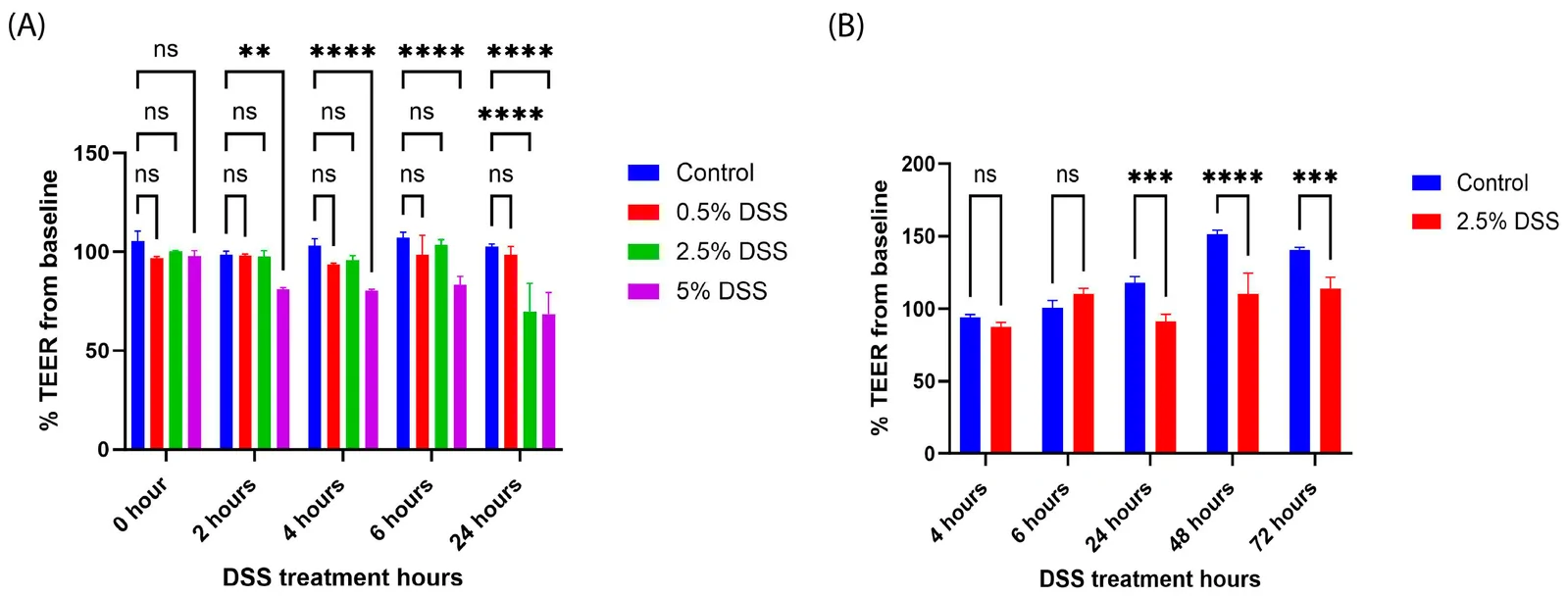
Effects of DSS treatment on epithelial barrier integrity in Caco-2 monolayer. Changes in epithelial barrier integrity across treatment conditions and time periods were evaluated by transepithelial electrical resistance (TEER) measurements, expressed as a percentage of baseline TEER. (**A**) Caco-2 monolayers were treated with increasing DSS concentrations (0.5%, 2.5%, 5%(*w*/*v*)) and TEER was measured at 0, 2, 4, 6, and 24 h. Statistical comparisons were performed between each DSS-treated group and the untreated control at the corresponding time point using two-way ANOVA with Bonferroni’s multiple comparisons test. (**B**) TEER measurements of Caco-2 monolayers treated with 2.5%(*w*/*v*) DSS over a prolonged period (4–72 h). Statistical comparisons were performed between the 2.5% DSS-treated and the untreated control groups at each corresponding time point using two-way ANOVA with Bonferroni’s multiple comparisons test. For both panels, data are presented as mean ± SD from three independent biological experiments (*n* = 3). A *p*-value < 0.01 (**), *p*-value < 0.001 (***), *p*-value < 0.0001 (****) are considered statistically significant; (ns) represents non-significant.

Differentiated Caco-2 monolayers were treated with increasing concentrations of DSS (0.5%, 2.5%, and 5%) *w*/*v* to establish an in vitro model of epithelial barrier disruption, and barrier integrity was assessed over time using transepithelial electrical resistance (TEER) measurements. The concentration gradient was selected based on previously reported DSS concentrations used in Caco-2 epithelial injury models. Previous studies evaluating DSS concentrations ranging from 0.05% to 5% demonstrated limited changes in epithelial permeability at lower concentrations, measurable barrier disruption at intermediate concentrations around 2–2.5%, and more pronounced loss of barrier integrity at higher concentrations of 3–5% [25,29]. Based on these findings, 0.5%, 2.5%, and 5% DSS were selected to represent a range of DSS exposures and to identify a concentration that produced sustained barrier dysfunction without excessive cytotoxicity. At early time points, DSS treatment didn’t show significant differences in TEER, indicating that short-term exposure did not immediately impact epithelial barrier integrity. However, DSS administration caused a dose-dependent drop in TEER at subsequent time points, with the effects being more pronounced at higher concentrations. Specifically, while 0.5%(*w*/*v*) and 2.5%(*w*/*v*) showed minimal changes in TEER reduction after 6 h of DSS treatment, the 5% DSS treatment group showed a significant reduction in TEER values from baseline. After 24 h, however, both the 2.5%(*w*/*v*) and 5%(*w*/*v*) concentrations exhibited statistically significant reductions in TEER values from baseline relative to the control. The 5%(*w*/*v*) concentration resulted in the most substantial barrier disruption; however, the extent of damage was too severe for a moderate injury model. The 2.5%(*w*/*v*) concentration, on the other hand, caused a controlled and reproducible degree of epithelial injury consistent with the parameters we were aiming for moderate damage required for the subsequent improvement in barrier function (Figure 1A).

To further evaluate the suitability of a 2.5%(*w*/*v*) concentration of DSS as a barrier-disrupting agent, TEER was monitored across a prolonged period up to 72 h. Consistent with earlier findings in Figure 1A, no significant changes were reported at earlier time points, i.e., 4 h and 6 h. A statistically significant reduction in TEER was observed after 24 h of DSS treatment, which was further accentuated even after 48 h, suggesting progressive and time-dependent impairment of epithelial barrier integrity. Similarly, at 72 h, percentage TEER values remained significantly reduced relative to the baseline, suggesting that the barrier disruption induced by 2.5%(*w*/*v*) DSS was sustained rather than transient throughout the observed period (Figure 1B).

### 2.2. 2.5%(w/v) DSS Treatment Induces Epithelial Barrier Dysfunction Without Causing Significant Cytotoxicity

**Figure 2:**
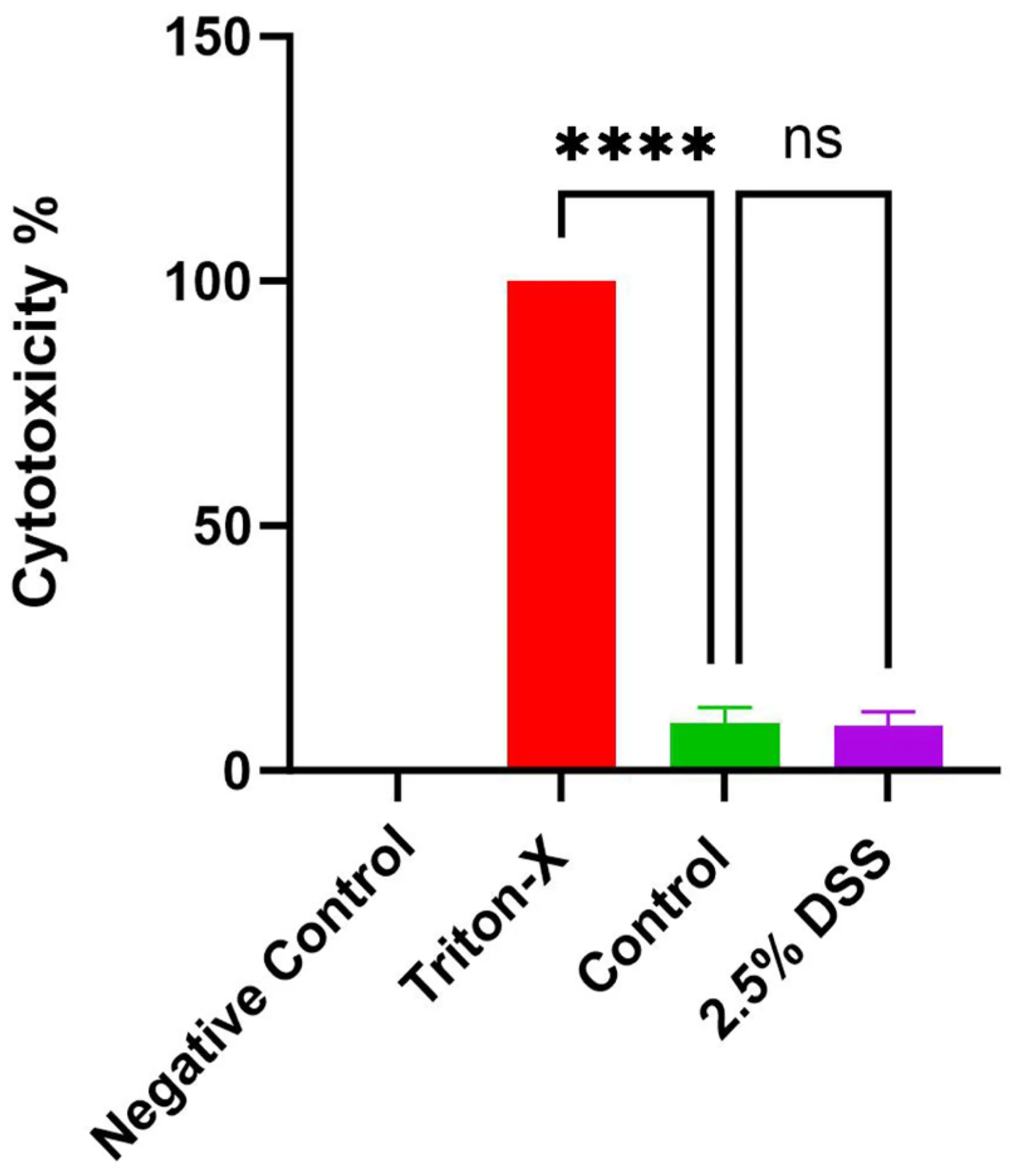
LDH Cytotoxicity Assay post 24 h of DSS treatment in Caco-2 cell monolayer. This figure illustrates cytotoxicity, which was quantified as the percentage of Lactate Dehydrogenase (LDH) release in Caco-2 cells following treatment with 2.5%(*w*/*v*) DSS for 24 h. The negative control represents background LDH levels present in the culture medium under untreated conditions, while Triton X-100 served as a positive control to induce maximum cytotoxicity. Cytotoxicity (%) was calculated relative to the maximum LDH release elicited by Triton-X 100. Each group contained three biological replicates (n = 3), and the data are represented as mean ± SD. Statistical comparisons were performed between the negative control and Triton X-100 groups and between the untreated control and 2.5%(*w*/*v*) DSS-treated groups using two-way ANOVA with Bonferroni’s multiple comparisons test. A *p*-value < 0.0001 (****) is considered statistically significant; (ns) represents non-significant.

To determine whether 2.5%(*w*/*v*) DSS treatment for 24 h compromised cell viability in Caco-2 cell monolayers, we performed a Lactate Dehydrogenase Assay (LDH Assay). Since LDH is a stable cytosolic enzyme released only when cell membranes are leaky or cells are damaged, this assay was suitable for assessing the cytotoxicity of our treatment. For the negative control, cell-free culture medium was included to determine background LDH levels, while cells treated with Triton-X 100 were used as a positive control to induce complete lysis of the cells and define the upper limit of LDH release. LDH release was measured following DSS treatment. No statistically significant LDH release was observed in the DSS-treated group compared to the untreated control, indicating that 2.5% DSS exposure did not induce measurable cytotoxicity after 24 h of treatment. In contrast, Triton-X 100 treatment caused a significant increase in LDH release that confirmed assay sensitivity and validated the integrity of the experimental setup (Figure 2). Collectively, these results suggest that the reduction in TEER observed in Figure 1 was due to increased epithelial barrier permeability rather than overt cell death, supporting the use of 2.5% DSS treatment for 24 h to induce a moderate, non-lethal barrier-injury model.

### 2.3. DSS Treatment Significantly Upregulates Oxidative Stress Marker NOX1 Expression in Caco-2 Cells

**Figure 3:**
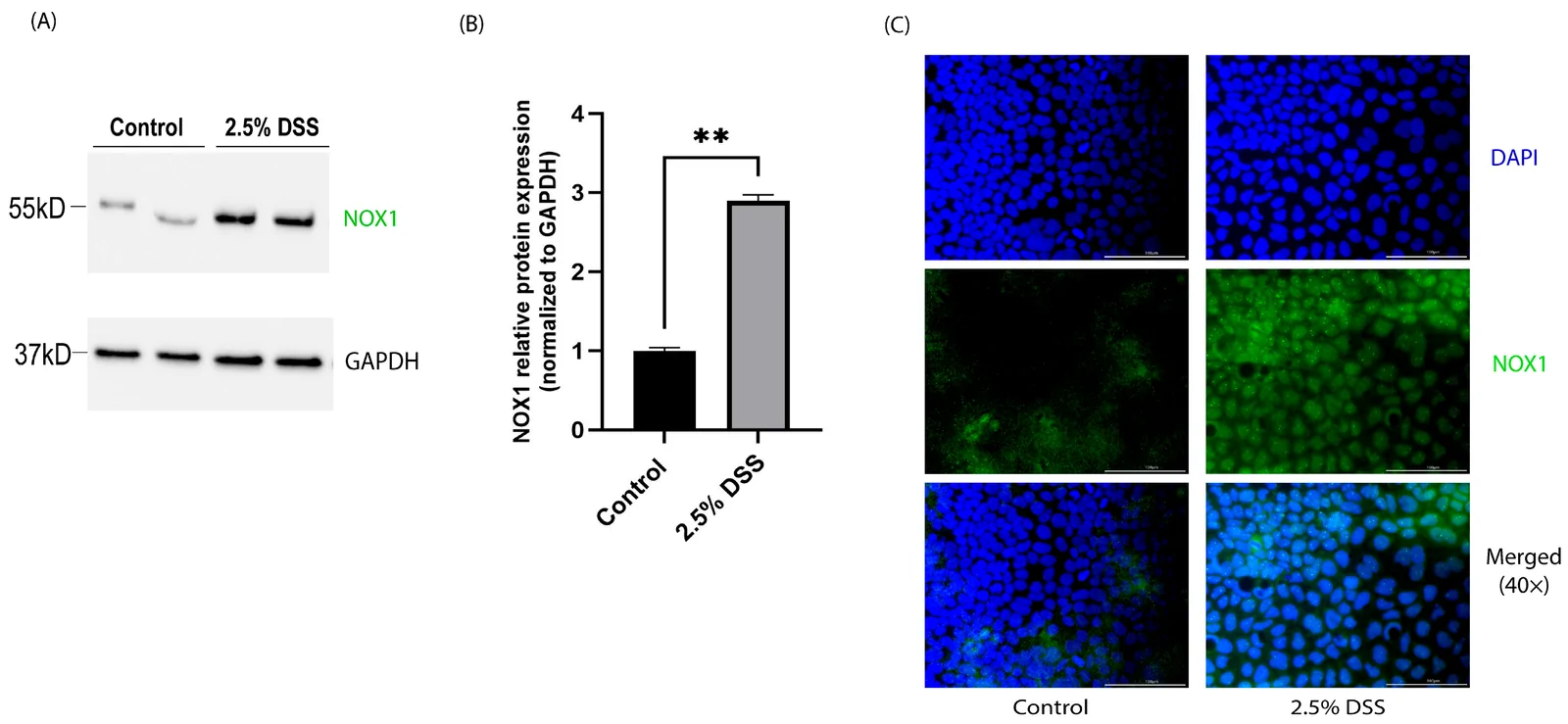
NOX1 protein expression analysis in DSS-treated Caco-2 cells using Western blot analysis and protein immunostaining. (**A**) Representative Western blot showing NOX1 protein expression in untreated control and 2.5%(*w*/*v*) DSS-treated Caco-2 cells for 24 h, with GAPDH serving as a loading control. (**B**) Densitometric quantification of NOX1 protein expression normalized to GAPDH and expressed relative to the untreated control. (**C**) Representative immunocytochemistry images of untreated control and 2.5%(*w*/*v*) DSS-treated Caco-2 cells showing NOX1 expression (green) and DAPI-stained nuclei (blue). Images were acquired at 40× magnification; scale bar = 100 μm. Merged pictures show the subcellular distribution and localization of NOX1 within the epithelial monolayer. Experiments were performed using three independent biological replicates (n = 3). Data are expressed as mean ± SD. Statistical comparison for densitometric analysis was performed between the untreated control and 2.5%(*w*/*v*) DSS-treated groups using an unpaired two-tailed *t*-test. A *p*-value < 0.01 (**) is considered statistically significant.

To evaluate oxidative stress post-treatment with DSS on Caco-2 cell monolayer after 24 h, oxidative stress marker NOX1 was quantified using Western Blot analysis and fluorescent immunocytochemistry. Western blot analysis revealed statistically significant upregulation of NOX1 protein expression in DSS-treated cells compared with the untreated control (Figure 3A). When densitometric quantification of the band intensities was performed, normalized to the housekeeping control GAPDH, it further corroborated the findings observed in Figure 3A, confirming a significant upregulation in NOX1 protein levels following DSS exposure in Caco-2 cells (*p*-value < 0.01, n = 3, Figure 3B).

Fluorescent immunostaining using antibodies against NOX1 was performed to validate the above findings. The DSS-treated group exhibited a marked increase in green fluorescence intensity compared to the untreated control. DAPI counterstaining was used to confirm nuclear integrity and uniform cell distribution across the cell monolayer (blue channel). Merged images of these blue and green channels further highlight the enhanced NOX1 expression in response to DSS treatment. Collectively, these findings suggest that DSS treatment induces a significant upregulation of NOX1 protein expression in Caco-2 cells, which is consistent with the activation of NADPH oxidase-mediated oxidative stress pathways in the intestinal epithelium [30].

### 2.4. Comparison of DSS-Induced ROS with Mycobacterium avium Subspecies Paratuberculosis (MAP) Induced ROS Production in Caco-2 Cells

**Figure 4:**
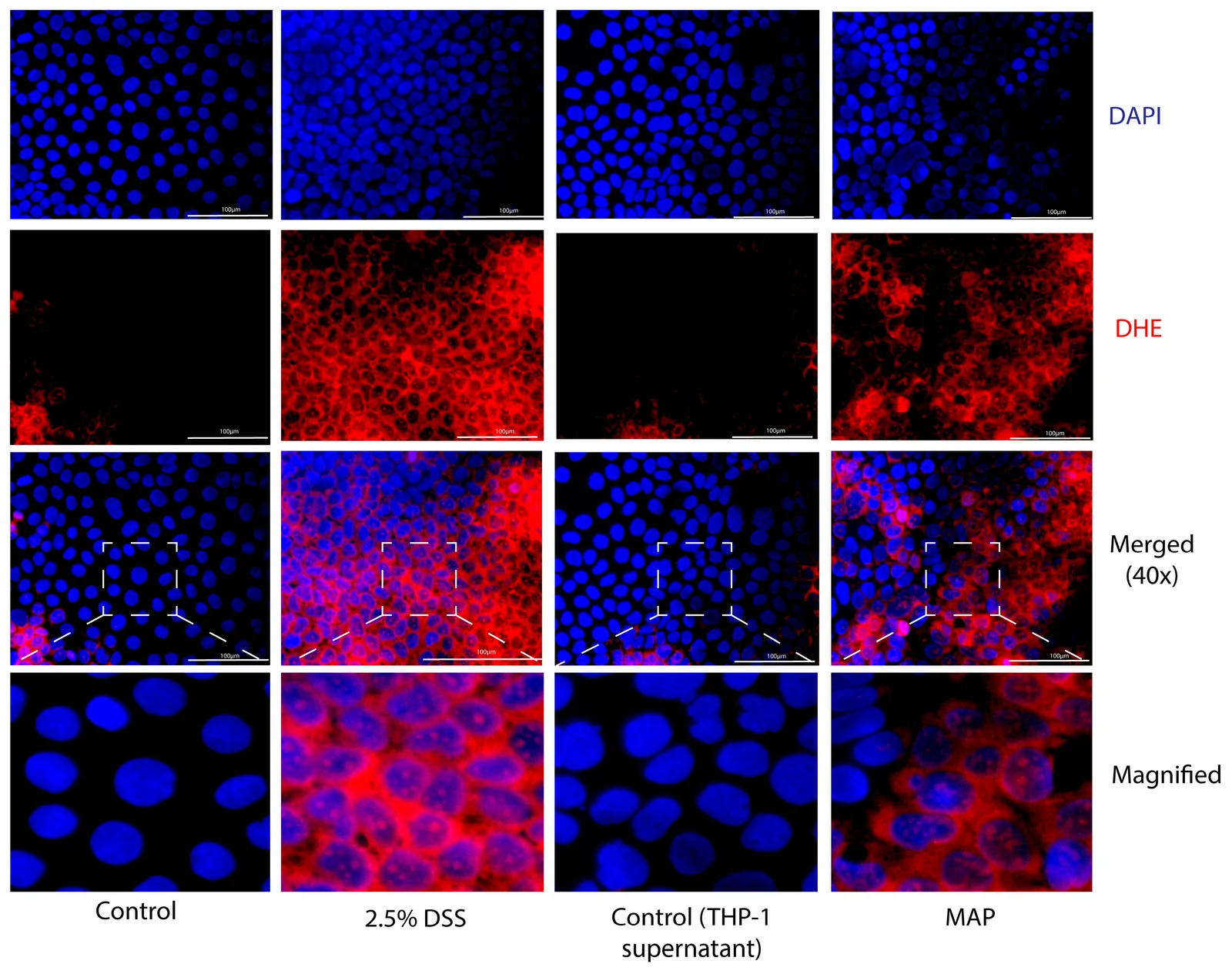
ROS production in DSS-treated Caco-2 cells compared with MAP inflammatory response. Representative microscopy images of Caco-2 cells under untreated control, 2.5%(*w*/*v*) DSS, THP-1 control supernatant, and MAP-infected THP-1 conditioned media conditions. Red fluorescence represents DHE staining of intracellular ROS production, whereas DAPI-stained nuclei are represented by blue fluorescence. Merged images show the localization of intracellular ROS. Images were taken at 40× magnification, and they were also digitally magnified (the area indicated by dashed boxes) to better visualize ROS distribution. Scale bar = 100 μm. Each group contains three independent biological replicates (n = 3), and representative images are shown.

To confirm the induction of oxidative stress within the DSS-treated Caco-2 epithelial injury model, intracellular reactive oxygen species (ROS) production was measured using dihydroethidium (DHE) staining. *NOX1* upregulation is generally associated with superoxide and ROS production. Therefore, this experiment was designed to validate whether DSS treatment led to functional oxidative stress at the cellular level. To further substantiate the model, ROS production in DSS-treated cells was compared against a well-established inflammatory model using *Mycobacterium avium* subspecies *paratuberculosis* (MAP). In this comparative model, THP-1 macrophages were infected with MAP, and the resulting conditioned media were used to treat Caco-2 cell monolayers as positive inflammatory control. DHE staining revealed low basal ROS levels in the untreated control cells, whereas there was a significant elevation in red fluorescence intensity, indicating increased intracellular ROS production. Similarly, when Caco-2 cells were exposed to conditioned medium derived from MAP-infected THP-1 macrophages, a robust DHE fluorescence was exhibited, which confirmed substantial oxidative stress induction. Conversely, when Caco-2 cells were treated with conditioned medium from non-infected THP-1 cells, this did not result in a noticeable increase in ROS levels, which confirms the specificity of the inflammatory response (Figure 4).

### 2.5. Pore-Forming Protein (Claudin-2) Expression Is Upregulated in Caco-2 Cell Line Following DSS Treatment

**Figure 5:**
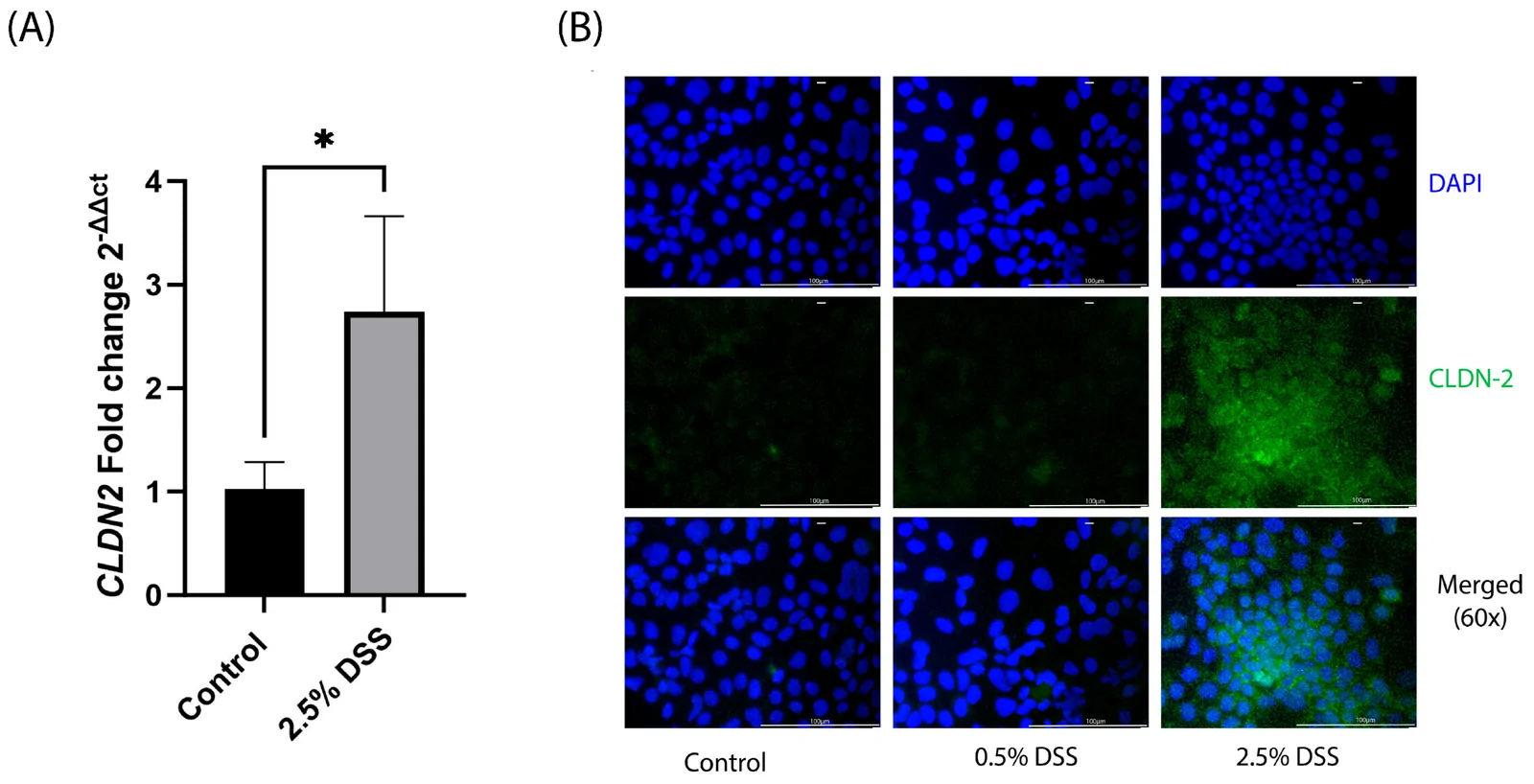
*CLDN2* gene expression and protein immunostaining in DSS-treated Caco-2 cells. (**A**) *CLDN2* mRNA expression in untreated control and 2.5%(*w*/*v*) DSS-treated Caco-2 cells was quantified using 2^−Δ∆Ct^ method and was normalized to GAPDH, which served as a housekeeping gene. Statistical comparison was performed between the untreated control and 2.5%(*w*/*v*) DSS-treated groups using an unpaired two-tailed *t*-test. (**B**) Representative fluorescence microscopy images of Caco-2 cells under untreated control, 0.5%(*w*/*v*) DSS, and 2.5%(*w*/*v*) DSS treatment conditions acquired at 60× magnification. Green fluorescence denotes immunoreactivity against Claudin-2 protein, whereas blue corresponds to nuclei counterstained with DAPI. Each experimental group was performed in three biological replicates, and data are expressed as mean ± SD. Statistical significance was defined as *p*-value < 0.05 (*).

Claudin-2(CLDN2) is a pore-forming tight junction protein that is expressed in intestinal epithelial cells and is responsible for facilitating the paracellular transport of water and monovalent cations (Na+, K+). Under inflammatory conditions, *CLDN2* expression is known to be upregulated transcriptionally and has been widely associated with increased epithelial barrier permeability and compromised tight junction integrity. In the present study, Claudin-2 expression was assessed at both the protein and mRNA levels using quantitative real-time PCR (qRT-PCR) and immunocytochemistry (ICC) in Caco-2 cells treated with DSS for 24 h. qRT-PCR analysis showed a statistically significant 2.677- fold upregulation of *CLDN2* mRNA expression in 2.5% DSS-treated cells compared to the untreated control (*p*-value < 0.05, Figure 5A). This finding was further validated with immunofluorescence analysis using anti-Claudin-2, and representative images are shown in Figure 5B. Claudin-2 expression was observed to be more pronounced with increasing DSS concentration. Immunofluorescence analysis revealed that 0.5% DSS treatment did not result in any noticeable change in Claudin-2 expression as compared to control cells. The 0.5% DSS condition was included as a lower-exposure condition to determine whether alterations in Claudin-2 were detectable at a milder level of epithelial injury. In contrast, 2.5% DSS resulted in a marked increase in Claudin-2 fluorescence intensity, which supports qRT-PCR results and indicates enhanced Claudin-2 expression post-2.5%(*w*/*v*) DSS exposure after 24 h on Caco-2 cell monolayers. The 5% DSS condition was not carried forward for this analysis because the initial TEER experiments demonstrated a pronounced loss of barrier integrity at this concentration.

### 2.6. DSS Treatment Reduces IL-6 Secretion in Caco-2 Cells

**Figure 6:**
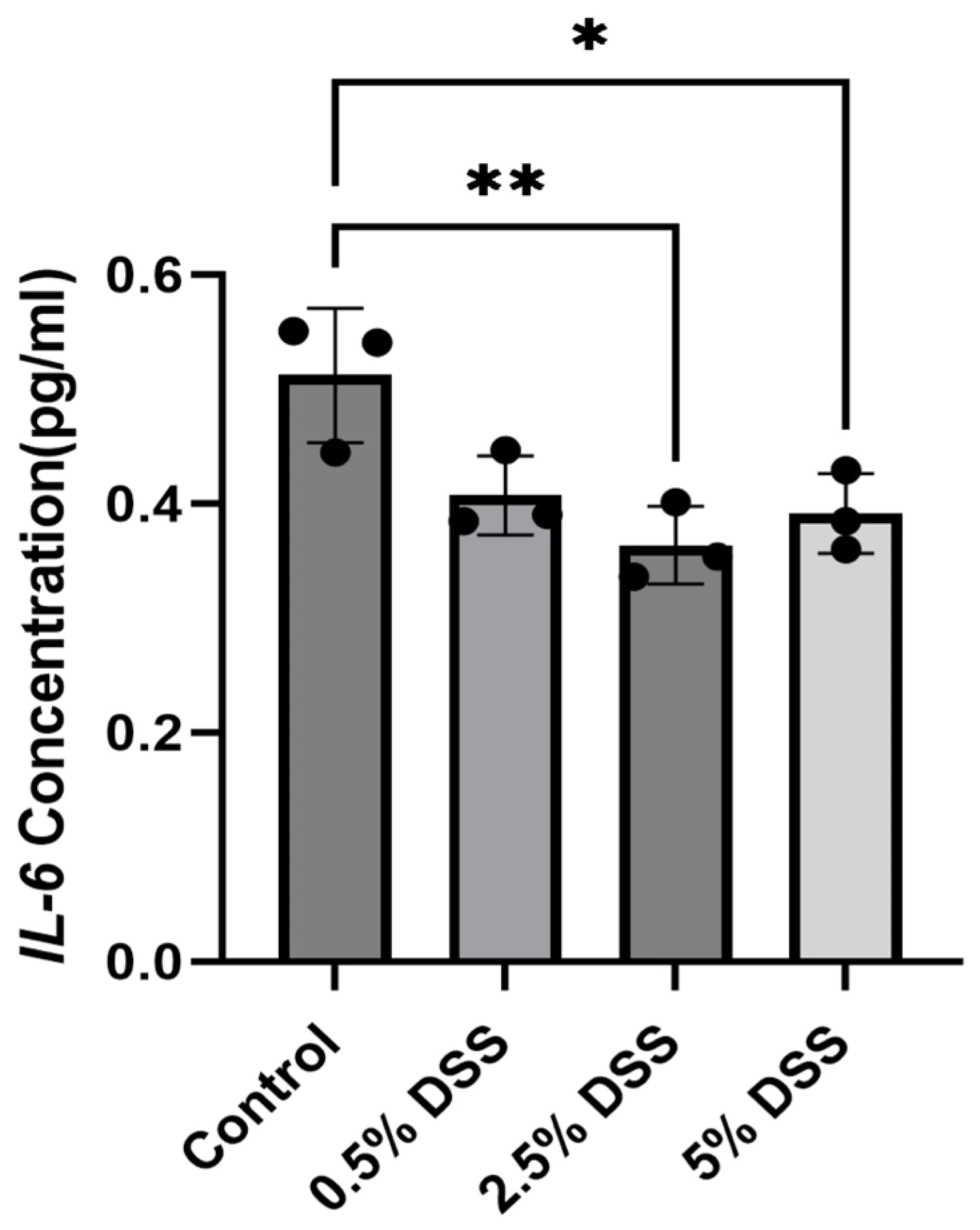
IL-6 secretion levels after 24 h of treatment with varying doses of DSS. IL-6 levels were measured (pg/mL) in Caco-2 cell culture supernatants following treatment with varying concentrations of DSS 0.5%(*w*/*v*), 2.5%(*w*/*v*), 5%(*w*/*v*) for 24 h using ELISA. Three independent biological experiments were performed (n = 3), with technical replicates used for ELISA measurements within each experiment. Data are presented as mean ± SD. Statistical comparisons were performed between the untreated control and the indicated DSS-treated groups using ordinary one-way ANOVA followed by Bonferroni’s multiple-comparisons test. A *p*-value < 0.05 (*) and *p*-value < 0.01 (**), are considered statistically significant.

Supernatants were collected from Caco-2 cells after 24 h of exposure to varying concentrations of DSS, and IL-6 levels were analyzed using ELISA. As shown in Figure 6, treatment with 0.5%(*w*/*v*) Treatment with 0.5%(*w*/*v*) DSS resulted in a modest reduction in IL-6 secretion to 0.407 ± 0.034 pg/mL, while a greater reduction was observed following treatment with 2.5%(*w*/*v*) DSS (0.364 ± 0.034 pg/mL). IL-6 secretion also remained lower following treatment with 5%(*w*/*v*) DSS (0.391 ± 0.035 pg/mL) (Figure 6). Overall, these findings indicate that DSS exposure was associated with reduced IL-6 secretion from Caco-2 cells under the conditions tested, although the reduction was not strictly dose-dependent.

### 2.7. Low Dose of rIL-6 Promotes Epithelial Barrier Restoration During Continued DSS Exposure

**Figure 7:**
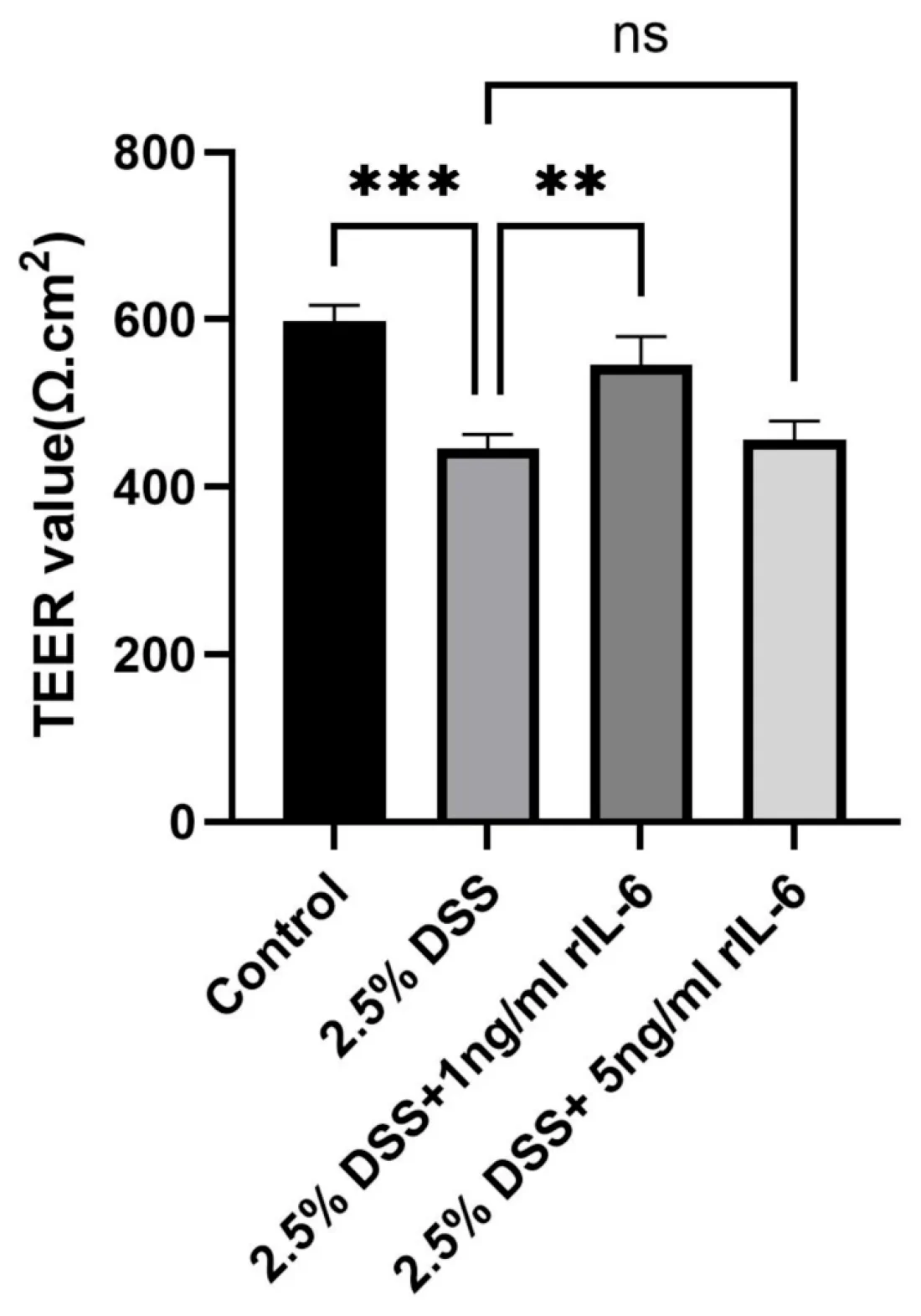
Effect of graded doses of rIL-6 on TEER in a DSS-treated cell monolayer. TEER measurements of Caco-2 monolayers treated with lower and higher doses of recombinant IL-6 (1 ng/mL and 5 ng/mL) in 2.5%(*w*/*v*) DSS-treated cells for 24 h are shown in this figure. Experiments were performed using three independent biological replicates (n = 3). Data are presented as mean ± SD. Statistical comparisons were performed between the indicated treatment groups using one-way ANOVA followed by Bonferroni’s multiple-comparisons test. A *p*-value < 0.001 (**) and *p*-value < 0.0001 (***) are considered statistically significant, while ns represents non-significance.

Intestinal epithelial cells, Caco-2, were exposed to a low dose of 1 ng/mL and a higher dose of rIL-6, 5 ng/mL, during continued DSS exposure. A low dose of rIL-6 was used to mimic the protective or basal signaling, whereas higher doses represent inflammatory conditions, to study the dual role of IL-6. The integrity of the epithelial barrier was assessed using TEER after rIL-6 administration during continued DSS exposure. As shown in Figure 6, treatment with a low dose of rIL-6 1 ng/mL, resulted in a significant increase (*p*-value < 0.01, Figure 7) in TEER value compared to the 2.5%(*w*/*v*) DSS treatment group, consistent with earlier findings, indicating partial restoration of barrier integrity. In contrast, treatment with a higher concentration of rIL-6 (5 ng/mL) did not result in significant improvement in TEER compared to the DSS-treated group (ns). These results suggest that a low dose of rIL-6 improves epithelial barrier function, while a higher concentration does not confer the same restorative effect, suggesting a concentration-dependent effect of rIL-6 in the maintenance of epithelial barrier integrity.

### 2.8. Effect of rIL-6 Concentration on NOX1 Expression During Continued DSS Exposure

**Figure 8:**
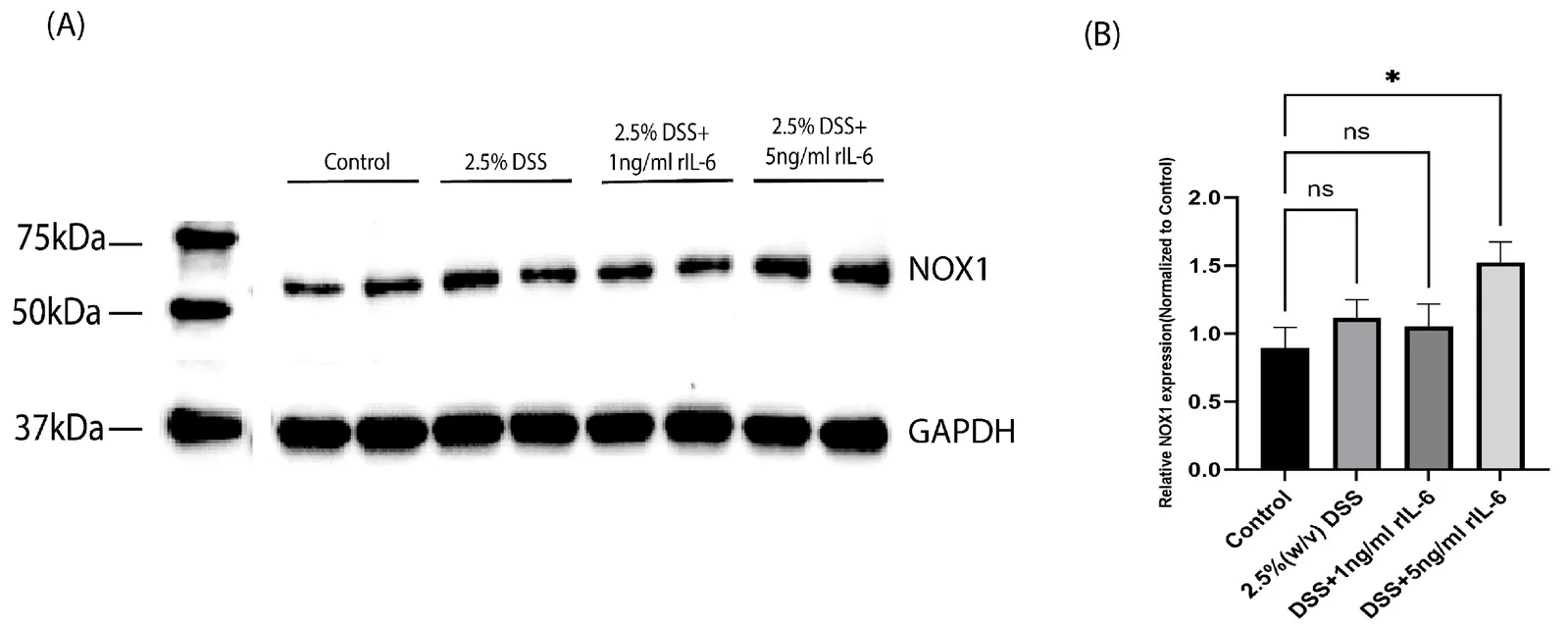
NOX1 protein expression in DSS-treated Caco-2 cells following recombinant IL-6 (rIL-6) treatment. (**A**) Representative Western blot showing NOX1 protein expression in untreated control, 2.5%(*w*/*v*) DSS-treated, and 2.5%(*w*/*v*) DSS+ recombinant IL-6 (1 ng/mL and 5 ng/mL) treatment conditions. GAPDH was used as a loading control. (**B**) Densitometric quantification of NOX1 expression normalized to GAPDH. Data are presented as mean ± SD. Experiments were performed with three independent biological replicates (n = 3). Statistical comparison for densitometric analysis was performed between the untreated control and indicated treatment groups using one-way ANOVA followed by Bonferroni’s multiple-comparisons test. A *p*-value < 0.05 (*) and ns (not significant) are indicated.

NOX1 protein expression was measured in DSS-treated Caco-2 cells following treatment with different concentrations of rIL-6 to evaluate its effect on NOX1 expression during epithelial injury. As shown in Figure 8A, exposure to 2.5%(*w*/*v*) DSS showed a trend toward increased NOX1 expression compared with the untreated control, consistent with the pattern observed in other experiments in this study. Addition of 1 ng/mL rIL-6 to DSS-treated cells did not significantly alter NOX1 expression compared with DSS treatment alone (ns). In contrast, treatment with 5 ng/mL recombinant IL-6 was associated with increased NOX1 expression; densitometric analysis normalized to GAPDH showed a significant increase compared with the untreated control (*p*-value < 0.05; Figure 8B). Overall, the Western blot analysis showed different patterns of NOX1 expression across the rIL-6 concentrations, with the 1 ng/mL treatment group remaining comparable to the untreated control, whereas significantly elevated NOX1 expression was observed with 5 ng/mL rIL-6.

### 2.9. Low Dose of rIL-6 Attenuates While High Dose IL-6 Enhances Claudin-2 Expression in DSS-Treated Caco-2 Cells

**Figure 9:**
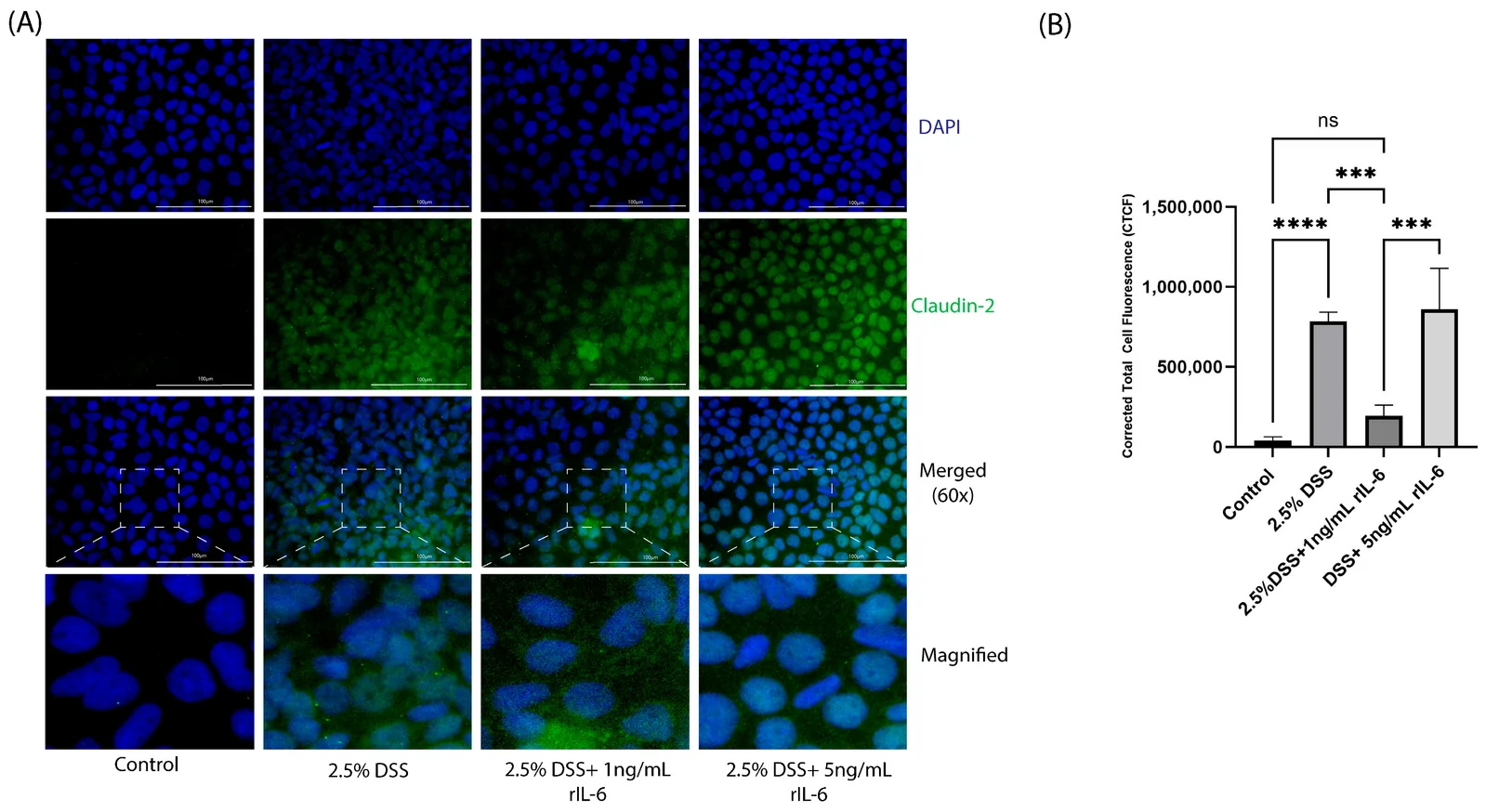
Claudin-2 protein expression in DSS-treated Caco-2 cells following recombinant IL-6 treatment. (**A**) Representative microscopy images of Caco-2 cells under untreated control, 2.5%(*w*/*v*) DSS, 2.5%(*w*/*v*) DSS + recombinant IL-6 (1 ng/mL and 5 ng/mL) treatment conditions. Green fluorescence signifies staining with anti-Claudin-2, while blue represents the nuclei of each cell (stained with DAPI). Merged images are shown to visualize protein localization. In the last row, the images were digitally magnified (area highlighted with a dashed box) to better visualize the cells. Images were acquired at 60× magnification. Scale bar = 100 μm. (**B**) Quantification of Claudin-2 expression using corrected total cell fluorescence (CTCF). Data are presented as mean ± SD. Statistical comparisons were performed between the 2.5% DSS-treated group and the indicated treatment groups, as well as between the untreated control and the 2.5% DSS + 1 ng/mL IL-6 group, using ordinary one-way ANOVA followed by Bonferroni’s multiple-comparisons test. A *p*-value < 0.001 (***) and *p*-value < 0.0001 (****) were considered statistically significant; ns, not significant.

Using immunocytochemistry and fluorescence measurement, we assessed the expression of the pore-forming tight junction protein Claudin-2 (CLDN-2) to better understand how IL-6 regulates epithelial barrier integrity after DSS-induced damage. Treatment with 2.5% DSS resulted in a significant increase in Claudin-2 fluorescence, as shown in Figure 9A, in line with earlier findings, suggesting increased expression of this pore-forming protein. A discernible decrease in Claudin-2 fluorescence was observed when 1 ng/mL rIL-6 was administered to DSS-treated cells, as opposed to DSS alone, demonstrating a possible protective impact of a low dose of rIL-6 on tight junction integrity. On the contrary, Claudin-2 expression increased significantly after treatment with 5 ng/mL rIL-6, comparable to or slightly exceeding levels observed in DSS-treated cells (Figure 9A). These findings were supported by quantitative analysis of corrected total cell fluorescence (CTCF) (Figure 9B), which further confirmed these observations showing a significantly lower Claudin-2 expression in 1 ng/mL treated group than the DSS-treated group (*p*-value < 0.001, Figure 9B), while the 5 ng/mL rIL-6 group had significantly higher Claudin-2 levels than the 1 ng/mL group (*p*-value < 0.001, Figure 9B). There was no significant difference between the 5 ng/mL rIL-6 group and DSS (ns). These results suggest that rIL-6 regulates tight junctions in a dose-dependent manner, with lower amounts potentially reducing epithelial permeability and higher concentrations further contributing to barrier disruption through increased expression of Claudin-2.

### 2.10. Butyrate Restores Epithelial Barrier Function During Continued DSS Exposure in Caco-2 Cells

**Figure 10:**
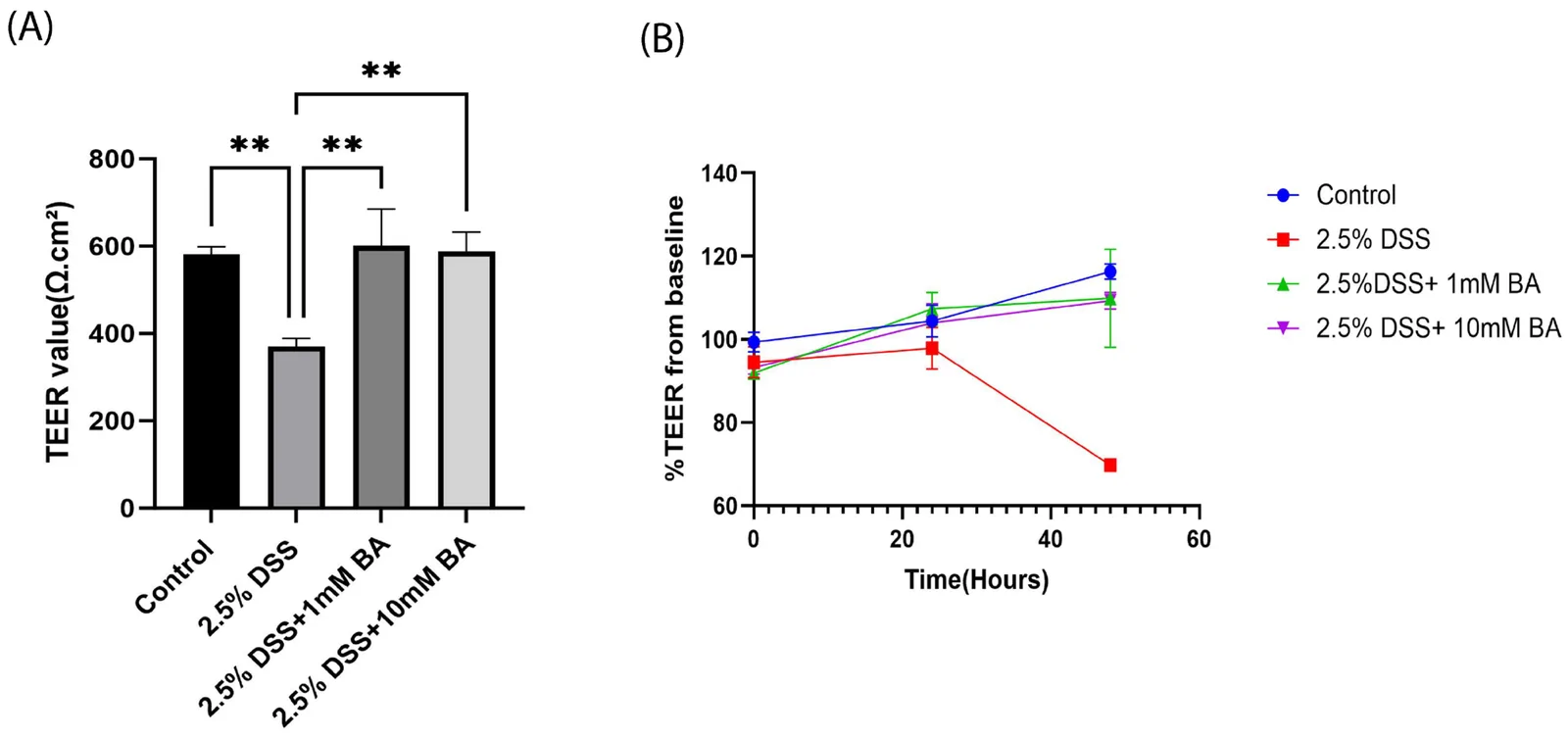
Effect of butyric acid on TEER in DSS-treated Caco-2 cell monolayer. (**A**) TEER measurements (Ω.cm^2^) in untreated control, 2.5%(*w*/*v*) DSS-treated, and DSS-treated Caco-2 monolayers supplemented with butyric acid (1 mM or 10 mM) following 24 h treatment. (**B**) Time-course analysis of TEER, expressed as a percentage (%) from baseline following butyric acid treatment during continued DSS exposure. Experiments were performed using three independent biological replicates (n = 3). Data are presented as mean ± SD. Statistical comparisons were performed between the 2.5% DSS-treated group and the indicated butyric acid treatment groups using ordinary one-way ANOVA followed by Bonferroni’s multiple-comparisons test. A *p*-value < 0.01 (**) was considered statistically significant.

We measured the impact of butyric acid (BA) on TEER in DSS-treated Caco-2 cells to assess the potential of microbial metabolites in restoring epithelial barrier integrity. Barrier disruption was confirmed, as treatment with 2.5%(*w*/*v*) DSS led to a significant drop in TEER compared to control (~600 vs. ~350 Ω.cm^2^; *p*-value < 0.01), as seen in Figure 10A. Post-treatment with 1 mM BA restored TEER levels close to control (~600 Ω.cm^2^; *p*-value < 0.01). Similarly, treatment with 10 mM BA also improved TEER compared to DSS-treated cells (~580 Ω.cm^2^; *p*-value < 0.01), indicating improvement of barrier function (Figure 10A). TEER was further evaluated over time following BA treatment to assess changes in epithelial barrier function during continued DSS exposure. As expected, DSS-treated cells showed a progressive decline in TEER with time, reaching approximately 70% of baseline (Figure 10B). On the other hand, TEER gradually increased over time in cells treated with 1 mM and 10 mM BA, reaching baseline levels. BA concentrations significantly improved epithelial barrier function during continued DSS exposure (*p*-value < 0.0001; Figure 10A). These findings suggest that BA promotes restoration of epithelial barrier integrity despite continued DSS exposure, with both concentrations showing barrier-restorative effects over time.

### 2.11. Butyric Acid Suppresses NOX1 Associated Oxidative Stress in DSS-Treated Caco-2 Cells

**Figure 11:**
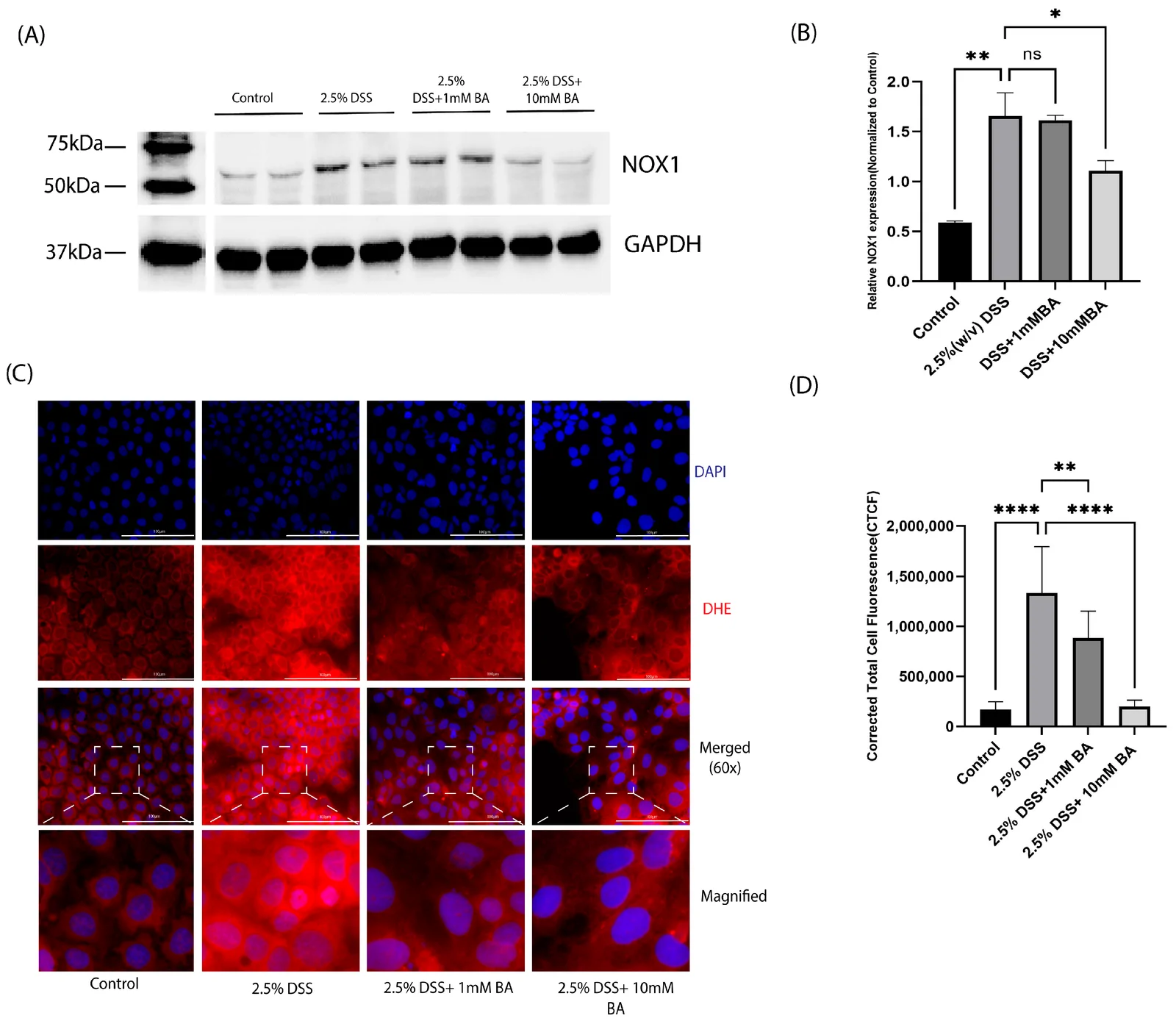
Effect of butyric acid on NOX1 protein expression and ROS production in DSS-treated Caco-2 cells. (**A**) Representative Western blot showing NOX1 protein expression in Caco-2 cells under control, 2.5%(*w*/*v*) DSS, and 2.5%(*w*/*v*) DSS+ butyric acid (1 mM and 10 mM) treatment conditions. GAPDH was used as a loading control. (**B**) Densitometric quantification of NOX1 expression normalized to GAPDH and expressed relative to the untreated control. Experiments were performed with three independent biological replicates (n = 3). Data are expressed as mean ± SD. Statistical comparison for densitometric analysis was performed between the 2.5% DSS-treated group and the untreated control using one-way ANOVA followed by Bonferroni’s multiple-comparisons test. (**C**) Representative fluorescence microscopy images of Caco-2 cells under control, 2.5%(*w*/*v*) DSS, and DSS+ butyric acid (1 mM and 10 mM) treatment conditions. Red fluorescence signifies DHE staining of reactive oxygen species (ROS), while blue represents the nuclei of each cell (stained with DAPI). Merged images are shown to visualize ROS localization. In the last row, the images were digitally magnified (area highlighted with a dashed box) to better visualize the cells. Images were acquired at 60× magnification. Scale bar = 100 μm. (**D**) Quantification of ROS levels using corrected total cell fluorescence (CTCF). Statistical comparisons were performed between the 2.5% DSS-treated group and the indicated treatment groups, as well as between the untreated control and 2.5% DSS-treated group, and between the 2.5% DSS-treated group and the 1 mM and 10 mM butyric acid treatment groups using ordinary one-way ANOVA followed by Bonferroni’s multiple-comparisons test. A *p*-value < 0.05 (*), *p*-value < 0.01 (**), and *p*-value < 0.0001 (****) were considered statistically significant; ns represents non significant.

We next assessed NOX1 protein expression by Western blot analysis in Caco-2 cells to further explore whether butyric acid affects oxidative stress during continued DSS exposure. In comparison to control cells, treatment with 2.5%(*w*/*v*) DSS clearly increased NOX1 expression, as shown in Figure 11A. Following DSS treatment, cells treated with 1 mM BA showed persistent NOX1 upregulation, shown by stronger band intensity comparable to the DSS treatment group, compared to the untreated control group, which was not significantly different from the DSS-treated group (ns), suggesting that oxidative stress signaling is not greatly reduced at this concentration. On the contrary, compared to cells treated with DSS, treatment with a higher dose of 10 mM BA led to a discernible decrease in NOX1 expression (Figure 11A). These findings were further supported by densitometric analysis normalized to GAPDH in Figure 11B, which demonstrates that 10 mM BA significantly decreased NOX1 expression in comparison to DSS (*p*-value < 0.05, Figure 11B), but the 1 mM BA group did not exhibit a significant difference (ns; Figure 11B). Dihydroethidium (DHE) staining was used to measure intracellular reactive oxygen species (ROS) levels and assess whether butyric acid (BA) reduces oxidative stress in DSS-treated epithelial cells. Untreated control cells showed little ROS generation, as seen in Figure 11C, whereas red fluorescence intensity increased significantly after treatment with 2.5%(*w*/*v*) DSS, indicating increased ROS levels after epithelial damage. Following DSS exposure, cells treated with 1 mM BA showed less DHE fluorescence than DSS-treated cells, which suggests a partial inhibition of ROS generation. Moreover, DHE fluorescence significantly decreased after treatment with 10 mM BA, reaching almost the same levels as observed in control cells. Quantitative analysis of corrected total cell fluorescence (CTCF) confirmed these observations, revealing that both 1 mM and 10 mM BA considerably lowered ROS levels in comparison to DSS (*p*-value < 0.0001; Figure 11D), with 10 mM showing a greater reduction than 1 mM (*p*-value < 0.01). These results show that butyric acid lowers DSS-induced oxidative stress in a dose-dependent manner, with NOX1 expression and ROS generation being more effectively suppressed at higher concentrations.

### 2.12. Butyric Acid Reduces Claudin-2 Expression in DSS-Treated Cells

**Figure 12:**
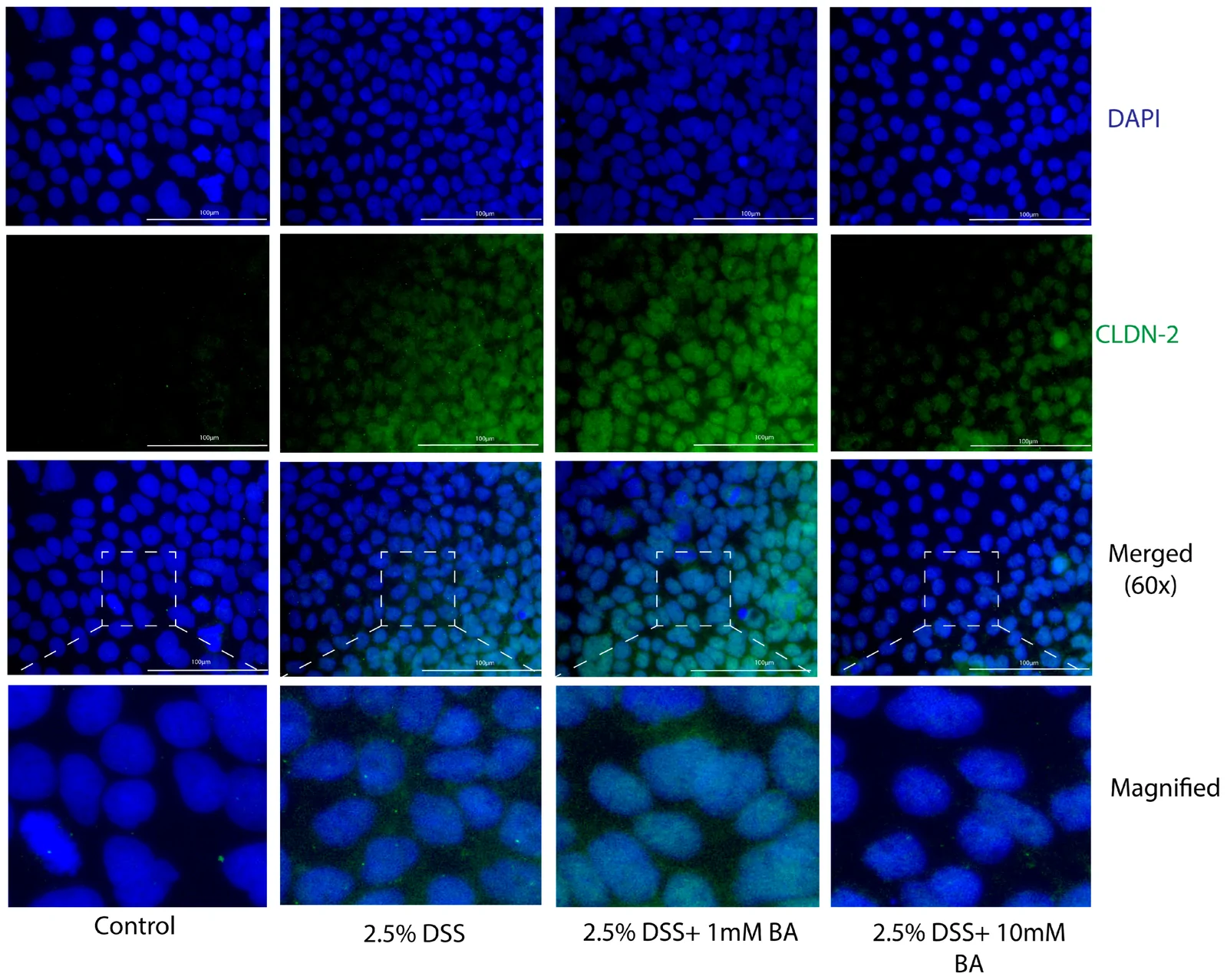
Claudin-2 protein expression in DSS-treated Caco-2 cells following butyrate treatment. This figure shows representative microscopy images of Caco-2 cells under control, 2.5%(*w*/*v*) DSS, and DSS + butyrate (1 mM and 10 mM) treatment conditions. Green fluorescence signifies staining with anti-Claudin-2, while blue represents the nuclei of each cell (stained with DAPI). Merged images are shown to visualize protein localization. In the last row, the images were digitally magnified (area highlighted with a dashed box) to better visualize the cells. Images were acquired at 60× magnification. Scale bar = 100 μm. Experiments were performed using three independent biological replicates (n = 3).

We then assessed the expression of the pore-forming protein Claudin-2(CLDN-2) to determine whether butyric acid (BA) affects tight junction integrity during continued DSS exposure using immunocytochemistry. As shown in Figure 12, control cells showed minimal green fluorescence, which indicates poor baseline expression of Claudin-2. The green fluorescence intensity significantly increased after treatment with 2.5% DSS, suggesting Claudin-2 was upregulated after epithelial damage. Similarly, a decrease in Claudin-2 fluorescence was observed in cells treated with 1 mM BA following DSS exposure compared to DSS-treated cells, confirming a partial restoration of tight junction integrity. Interestingly, Claudin-2 expression was significantly reduced after treatment with 10 mM BA, and fluorescence intensity approximated values observed in control cells. These findings suggest that butyric acid inhibits the dose-dependent expression of Claudin-2 caused by DSS, implying a reduction in paracellular permeability by downregulating the pore-forming tight junction protein Claudin-2, which aids in the restoration of epithelial barrier integrity.

### 2.13. Differential Effects of Butyrate and rIL-6 on NOX-1 Expression in DSS-Treated Cells

**Figure 13:**
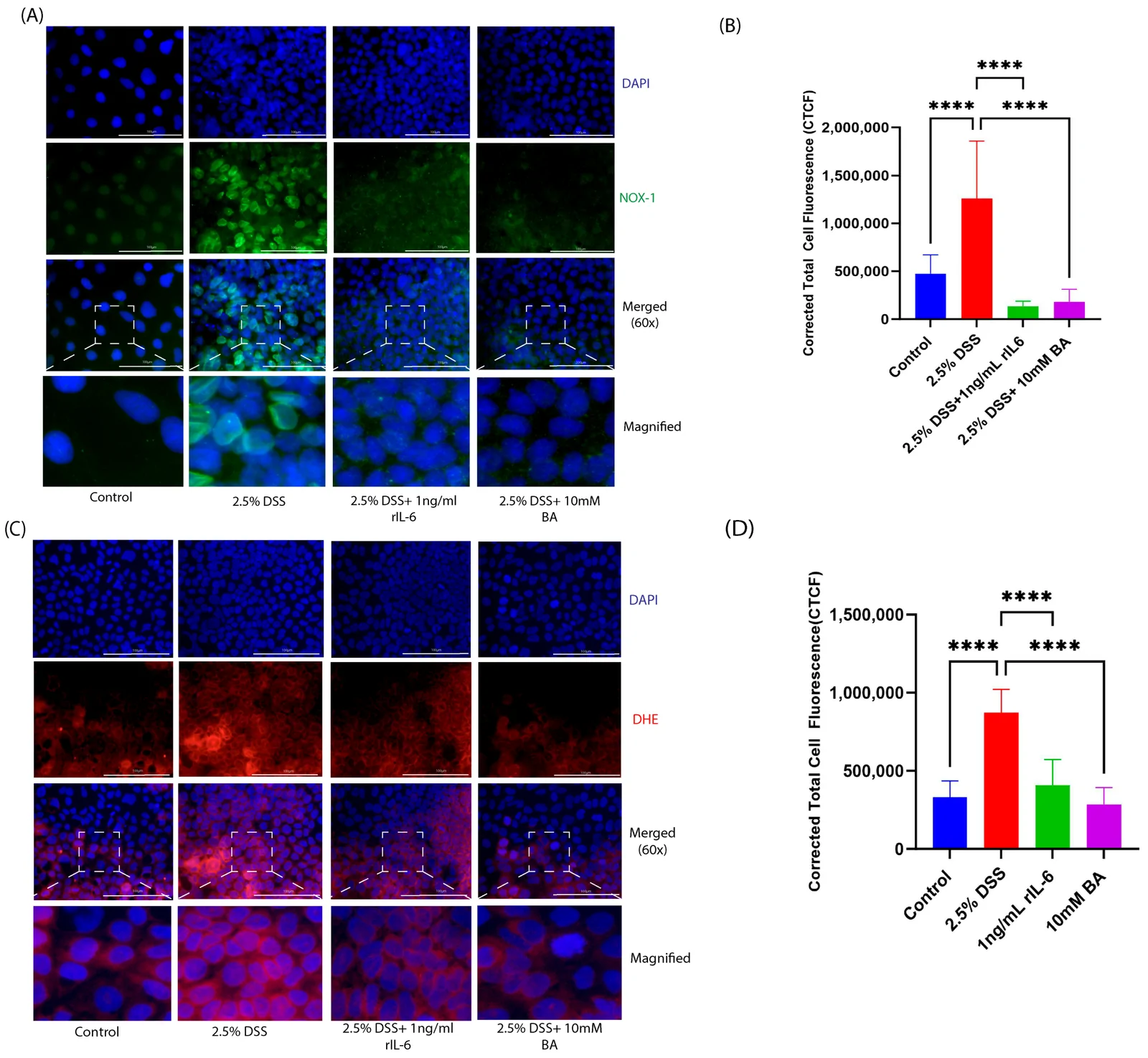
Effect of recombinant IL-6 and butyrate on NOX1 expression and ROS production in DSS-treated Caco-2 cells. (**A**) Representative immunocytochemistry images of Caco-2 cells under untreated control, 2.5%(*w*/*v*) DSS, 2.5% DSS + recombinant IL-6 (1 ng/mL), and 2.5% DSS + butyrate (10 mM) treatment conditions. NOX1 protein expression is shown in green, while nuclei were counterstained with DAPI (blue). Merged images show the distribution and localization of NOX1 within the epithelial monolayer. Images were acquired at 60× magnification, and selected regions indicated by dashed boxes were digitally magnified for better visualization. Scale bar = 100 µm. (**B**) Quantification of NOX1 expression using corrected total cell fluorescence (CTCF). Statistical comparisons were performed between the untreated control and 2.5% DSS-treated group and between the 2.5% DSS-treated group and the recombinant IL-6 (1 ng/mL) and butyrate (10 mM) treatment groups using ordinary one-way ANOVA followed by Bonferroni’s multiple-comparisons test. (**C**) Representative DHE staining images showing intracellular ROS production (red), with nuclei counterstained with DAPI (blue), under the same treatment conditions. Merged images show the distribution and localization of NOX1 within the epithelial monolayer. Images were acquired at 60× magnification. Scale bar = 100 µm. (**D**) Quantification of ROS levels using corrected total cell fluorescence (CTCF). Data are presented as mean ± SD. A *p*-value < 0.0001 (****) is considered statistically significant.

To compare the effects of cytokine signaling and microbial metabolites on oxidative stress, we used immunocytochemistry and DHE staining to evaluate NOX1 expression and ROS generation in DSS-treated Caco-2 cells after treatment with recombinant IL-6 (r-IL6) and butyrate (BA). As seen in earlier results, control cells had minimal baseline NOX1 fluorescence, as seen in representative immunocytochemistry images (Figure 13A), but treatment with 2.5%(*w*/*v*) DSS led to a significant increase in NOX1 expression and increased bright red fluorescence (Figure 13A,C), indicating oxidative stress induction and intracellular ROS generation. Compared to cells treated with DSS, treatment with 1 ng/mL rIL-6 decreased NOX1 fluorescence, suggesting attenuation of oxidative stress signaling. Similarly, NOX1 expression was significantly reduced after treatment with 10 mM BA, and fluorescence levels were comparable to those observed in control cells. NOX1 expression quantitative analysis (CTCF) supported these findings by demonstrating that DSS-treated cells had significantly higher levels of NOX1 than control cells (*p*-value < 0.0001; Figure 13B). When compared to DSS, both rIL-6 and BA treatments markedly decreased NOX1 expression (*p*-value < 0.0001; Figure 13B). DHE staining images showed low baseline ROS levels in control cells and a significant increase in red fluorescence after DSS treatment, which is consistent with our findings (Figure 13C). In comparison to cells treated with DSS, treatment with rIL-6 and BA decreased the intensity of ROS fluorescence, suggesting a reduction in oxidative stress. These findings were further confirmed by ROS level quantification with CTCF, which showed significant upregulation in ROS generation in cells treated with DSS, which was substantially reduced after treatment with both rIL-6 and BA (*p*-value < 0.0001; Figure 13D).

### 2.14. Differential Effects of Butyrate and Recombinant IL-6 on Claudin-2 Expression in DSS-Treated Cells

**Figure 14:**
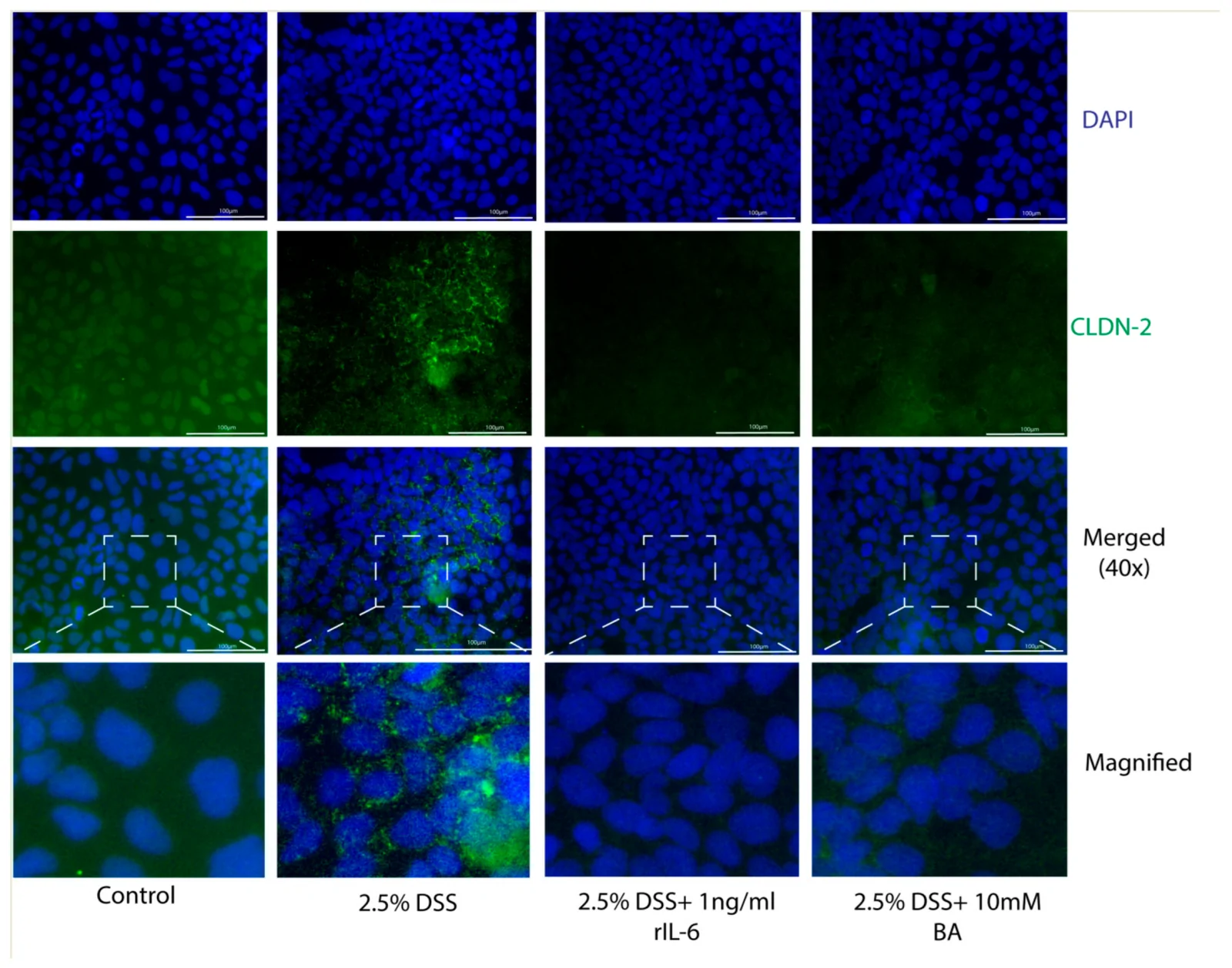
Effect of recombinant IL-6 and butyrate on Claudin-2 expression in DSS-treated Caco-2 cells. This figure shows representative immunocytochemistry images of Caco-2 cells stained for Claudin-2 (green) and nuclei (DAPI, blue) under control, 2.5%(*w*/*v*) DSS, and DSS+ recombinant IL-6 (1 ng/mL) and DSS + butyrate (10 mM) treatment conditions. Merged images are shown to visualize protein localization. In the last row, the images were digitally magnified (area highlighted with a dashed box) to better visualize the cells. Images were acquired at 60× magnification. Scale bar = 100 µm. Experiments were performed using three independent biological replicates (n = 3).

To compare the effects of cytokine signaling and microbial metabolites on pore-forming tight junction protein Claudin-2 regulation, we used immunocytochemistry in DSS-treated Caco-2 cells after treatment with recombinant IL-6 (r-IL6) and butyrate (BA). Figure 14 demonstrates that control cells exhibited low baseline CLDN-2 fluorescence. Treatment with 2.5%(*w*/*v*) DSS led to a marked increase in green fluorescence intensity, indicating upregulation of Claudin-2 and disruption of tight junction integrity. Exposure of DSS-treated cells to 1 ng/mL and 10 mM butyrate resulted in a notable decrease in Claudin-2 expression compared to DSS-treated cells, with fluorescence intensity approaching levels in control cells. Both rIL-6 and butyrate reduced DSS-induced Claudin-2 expression, indicating their potential roles in limiting epithelial barrier function.

## 3. Discussion

In the present study, we established and characterized a reproducible DSS-induced epithelial injury model using differentiated Caco-2 monolayers that mimics key features of intestinal barrier dysfunction and allows epithelial responses to be studied without the interference of external factors such as immune-mediated effects or systemic host responses. Using this model, we show that moderate DSS exposure leads to loss of epithelial barrier integrity, induces oxidative stress, and provides a suitable model for investigating epithelial barrier dysfunction and evaluating candidate barrier-restorative interventions in an epithelial-specific system.

To establish a robust in vitro epithelial injury model, one of the major aims of the study was to optimize an appropriate concentration of DSS that could maintain cell viability, thereby allowing subsequent evaluation of barrier-restorative responses during continued DSS exposure. DSS has been widely used to induce experimental colitis mainly in vivo because it directly disrupts epithelial integrity, increases intestinal permeability, and drives inflammatory responses through epithelial injury [21,31]. However, excessive DSS exposure often causes rapid and irreversible cell death, making it difficult to investigate epithelial repair mechanisms. Our findings demonstrated that although 5%(*w*/*v*) DSS produced a rapid decline in TEER, this concentration caused severe epithelial damage, as confirmed by the rapid decline in TEER, which was less suitable for evaluating barrier restoration during continued DSS exposure. In contrast, treatment with 2.5%(*w*/*v*) DSS resulted in a sustained reduction in TEER while causing minimal LDH release, indicating epithelial barrier dysfunction without causing excessive cytotoxicity. These findings suggest that the epithelial barrier integrity in the Caco-2 monolayer was disrupted due to exposure to a moderate concentration of DSS without causing non-specific cell death. Similar observations have been reported in previous in vitro DSS models, where moderate DSS concentrations produced reversible barrier dysfunction while preserving epithelial viability [25,32].

Oxidative stress plays a critical role in causing disruption of tight junction proteins, mitochondrial dysfunction, DNA damage, and activation of pro-inflammatory signaling pathways, as reported by several studies [33,34]. The most prevalent NADPH oxidase isoform expressed in intestinal epithelial cells, NOX1, is a significant generator of ROS during epithelial damage [8]. In both experimental colitis and human IBD, excessive NOX1 activation has been linked to oxidative damage, compromised epithelial barrier integrity, and increased intestinal permeability [35]. In the present study, increased NOX1 expression was accompanied by enhanced DHE fluorescence, confirming that DSS-induced epithelial injury was associated with elevated ROS generation. To further examine the physiological relevance of our model, oxidative stress responses following DSS exposure were compared with those induced by conditioned media from *Mycobacterium avium* subsp. *paratuberculosis* (MAP)-infected THP-1 macrophages, an inflammatory model previously established in our laboratory. Interestingly, both models produced comparable patterns of ROS generation, suggesting that despite the absence of immune cells, DSS-induced epithelial injury reproduces important oxidative features of inflammatory barrier dysfunction. This study is especially important since it shows that epithelial oxidative stress may be produced independently of immune cell-derived inflammatory mediators, allowing candidate barrier-restorative interventions to be evaluated in a controlled epithelial-specific setting. Previous studies have shown that ROS not only damages cellular macromolecules but also alters the expression and localization of tight junction proteins, thereby increasing epithelial permeability [36,37]. The concurrent increase in NOX1 expression and ROS production, together with reduced TEER and altered Claudin-2 expression, suggests an association between oxidative stress and epithelial barrier dysfunction following DSS exposure. These findings provide a basis for investigating whether recombinant IL-6 and butyrate can modulate epithelial barrier function and oxidative stress during continued DSS exposure.

Our ELISA results revealed that IL-6 levels were significantly downregulated with increasing concentrations of DSS. This was unexpected because IL-6 is associated with driving pro-inflammatory responses and has long been considered a marker for chronic inflammation, STAT3 activation, and immune cell recruitment [38,39,40]. As a result, there are several therapeutic strategies that have focused on completely blocking IL-6 signaling to reduce inflammation and tissue damage [41,42,43]. However, multiple studies have shown that the biological role of IL-6 is far more complex, and IL-6 is also essential for epithelial survival, mucosal repair, and tissue regeneration [44,45,46]. Notably, anti-IL-6 therapy has been reported to worsen mucosal healing, suggesting that a baseline level of IL-6 is required to maintain epithelial barrier integrity [47]. IL-6 can act through classical in which IL-6 binds the membrane bound IL-6 receptor whose expression is primarily limited to hepatocytes and immune cells, including intestinal epithelial cells, generally associated with regenerative and anti-inflammatory functions and trans-signaling which primarily drives chronic inflammation through the binding of IL-6 to the soluble IL-6 receptor that allows IL-6 to casacade downstream signaling through any gp130-expressing cell pathways, which have been associated with distinct biological responses and may provide context for the concentration-dependent effects observed in this study [13,39,48,49]. Based on our previous findings, we selected 1 ng/mL and 5 ng/mL rIL-6 to evaluate whether rIL-6 concentrations produce distinct effects on epithelial barrier function during continued DSS exposure, where rIL-6 concentrations up to 1 ng/mL had less apoptotic effect, whereas higher concentrations, such as 5 ng/mL, drove the inflammatory response. Therefore, 5 ng/mL rIL-6 was included as the higher experimental concentration for comparison with the response observed at 1 ng/mL. A low dose of rIL-6, 1 ng/mL improved epithelial barrier function during continued DSS exposure and was associated with modulation of NOX1 and reduced Claudin-2 expression, whereas the higher dose of 5 ng/mL did not produce the same restorative response. The established differences between classical and trans-signaling provide a possible biological context for the concentration-dependent effects observed in our model. However, the present study did not directly assess STAT3 activation or distinguish classical from trans-signaling, and therefore the specific pathway responsible for these effects cannot be determined from the current data. Rather, our findings demonstrate that different concentrations of rIL-6 produce distinct epithelial responses during continued DSS exposure, with 1 ng/mL promoting restoration of barrier function while 5 ng/mL did not produce the same restorative response. Although the present study did not measure STAT3 activation and therefore cannot selectively distinguish classical from trans-signaling, the concentration-dependent response observed in our epithelial model provides a strong basis for further investigation of these pathways. The use of a reductionist Caco-2 model allowed us to investigate the epithelial response to different rIL-6 concentrations without the interference of complex immune cell interactions, demonstrating distinct epithelial responses to the two rIL-6 concentrations evaluated in this study.

Beyond cytokine modulation, we evaluated the protective effects of butyrate, a short-chain fatty acid generated from the microbiome that is known to support intestinal epithelial health. Previous studies have shown that butyrate stabilizes tight junction proteins, enhances the integrity of the epithelial barrier, and prevents oxidative stress and inflammatory signaling [19]. Our TEER data demonstrated that both 1 mM and 10 mM butyrate improved epithelial barrier integrity during continued DSS exposure. While DSS-treated cells continued to exhibit declining barrier integrity, time-course analysis revealed a consistent improvement in TEER values in butyrate-treated cells. Crucially, butyrate treatment significantly reduced NOX1 expression, ROS production, and Claudin-2 expression in DSS-treated cells; 10 mM butyrate had a stronger effect. This is further supported by our previous work using a distinct in vitro epithelial injury model induced by conditioned media from MAP-infected macrophages, in which butyrate similarly reduced NOX1 expression and reversed Claudin-2 upregulation in Caco-2 monolayers [19]. According to the existing literature, the protective effects of butyrate are likely to be mediated by numerous complementary pathways rather than a single mechanism [50,51]. Butyrate is a primary energy source for colonocytes that is processed by β-oxidation in the mitochondria, which increases oxygen consumption and stabilizes hypoxia-inducible factor (HIF), which has been linked to regulating the integrity of the epithelial barrier [52,53]. Butyrate has also been shown to inhibit histone deacetylases, providing another mechanism through which it may regulate tight junction organization through regulation of the cytoskeletal protein synaptopodin [54]. Moreover, another study has shown that butyrate can reduce ROS generation while maintaining mitochondrial function following oxidative epithelial damage [18]. Together, these mechanisms suggest that the barrier-restorative effects observed in our study may be a combination of antioxidation activation, regulation of tight junction integrity, and support of epithelial energy metabolism rather than antioxidant activity alone. Future studies should also directly test the relative contribution of butyrate’s metabolic, epigenetic, and antioxidant actions, for example using HIF/PHD inhibitors, HDAC inhibitors, or direct antioxidant supplementation.

While the present Caco-2 monolayer model provides a controlled, epithelial-specific system for investigating barrier injury and repair, the model lacks the complexity of the in vivo intestinal environment. Therefore, our findings should be interpreted primarily as epithelial-specific responses to injury and potential barrier restorative interventions rather than a complete representation of the physiological response occurring in vivo. Important differences also exist between the present in vitro DSS Caco-2 model and in vivo DSS-induced colitis models. DSS concentrations and exposure conditions vary between different systems, and in vivo models include additional aspects like immune cell infiltration, cytokine signaling, stromal responses, and gut microbiota changes that cannot be replicated in a Caco-2 monoculture. Hence, further studies using three-dimensional intestinal organoids could better represent epithelial architecture, or epithelial-immune co-culture systems could help determine how immune cell interactions influence the responses observed in our model and would help bridge the gap between this reductionist platform and the full complexity of the in vivo intestinal microenvironment and would further strengthen the translational relevance of the present findings.

Consistent with previously reported DSS Caco-2 models, the present study demonstrated DSS-mediated epithelial barrier injury but expands on those previous models by adding oxidative stress pathways and investigating their connection to barrier dysfunction, tight junction remodeling, as well as distinct concentrations of rIL-6 and butyrate supplementation effects on the single-epithelial-injury framework. Importantly, using this model, we observed distinct effects of different rIL-6 concentrations on epithelial barrier function during continued DSS exposure. Low-dose rIL-6 (1 ng/mL) promoted epithelial restoration of barrier function during continued DSS exposure and was associated with modulation of NOX1 and reduced Claudin-2 expression, whereas a higher rIL-6 concentration (5 ng/mL) did not produce the same restorative response. These findings support the concept that the effects of IL-6 on the intestinal epithelium are dependent on its concentration and biological context rather than being uniformly pro-inflammatory or protective. In addition, the observed effects of rIL-6 and butyrate demonstrate their direct influences on epithelial barrier restoration during continued DSS exposure. Overall, this study provides a valuable, tractable platform for investigating intestinal epithelial injury and supports epithelial barrier repair as a possible barrier-restorative interventions that warrant further evaluation in more physiologically relevant models. In the same way, both 1 mM and 10 mM butyrate improved the function of the epithelial barrier, with 10 mM often eliciting a greater response along with a decrease in NOX1 expression, ROS generation, and Claudin-2 expression. These results show that the most efficacious concentrations tested in this DSS-induced Caco-2 epithelial injury model are 1 ng/mL rIL-6 and 10 mM butyrate. However, rather than being the best therapeutic dosages for IBD in humans, these concentrations should be understood as efficient experimental conditions within the current model.

## 4. Materials and Methods

### 4.1. Cell Culture and Maintenance

For cell culture experiments, we used the human monocytic cell line THP-1 and human enterocytic cell line Caco-2, both obtained from ATCC (Cat# TIB-202 and Cat# HTB-37, respectively; ATCC, Manassas, VA, USA). THP-1 monocytes were grown as suspension cells in T75 flasks (Thermo Scientific, Nunc, Waltham, MA, USA) using RPMI-1640 medium (Cat# 30-2001, ATCC, Manassas, VA, USA) supplemented with 0.005 mM 2-mercaptoethanol (Cat# 31350010, Gibco, Thermo Fisher Scientific, Waltham, MA, USA) and 10% fetal bovine serum (FBS; Cat# 30-2020, ATCC, Manassas, VA, USA). Cells were plated at a density of 5 × 10^5^ cells/mL in 12-well plates for general protein and molecular assays. After being seeded in 24-well plates, THP-1 monocytes were treated with 50 ng/mL phorbol 12-myristate 13-acetate (PMA) for 48 h to induce differentiation into macrophages. Following differentiation, the resultant THP-1 macrophages were infected with 1 × 10^2^ CFU/mL of *Mycobacterium avium* subspecies *paratuberculosis* (MAP) for 24 h. The conditioned media collected from these cells were used to treat intestinal cells for comparative analysis in downstream experiments. For intestinal epithelial experiments, Caco-2 cells were grown as adherent monolayers in T75 flasks (Thermo Scientific, Nunc, Waltham, MA, USA) with 20% fetal bovine serum (FBS; Cat# 30-2020, ATCC, Manassas, VA, USA) added to Eagle’s Minimum Essential Medium (EMEM; Cat# 30-2003, ATCC, Manassas, VA, USA). All cells were maintained in a humidified incubator at 37 °C in the presence of 5% CO_2_. Upon reaching approximately 80% confluency, Caco-2 cells were detached using Accutase (Cat# NC1793126, STEMCELL Technologies, Vancouver, BC, Canada), counted, and seeded for downstream assays. Cells were plated at a density of 3.5 × 10^5^ cells/mL in 12-well plates for general protein and molecular assays. For immunocytochemistry analysis, cells were seeded in 8-well chamber slides, and for barrier integrity experiments, cells were seeded onto polyester semipermeable Trans well inserts (0.3 µm pore size; Cat#662630, Griener Bio-One, Monroe, NC, USA) and maintained until a stable epithelial monolayer was formed. After 14–21 days, the cells were allowed to fully differentiate depending upon the assay, with media being changed every other day.

### 4.2. Dextran Sodium Sulfate (DSS) Treatment

Differentiated Caco-2 cell monolayers were exposed to Dextran sulfate sodium salt (DSS; Cat#42867, Sigma-Aldrich, St. Louis, MO, USA; Mr ~40,000). To obtain the required working concentrations, DSS was made fresh for every experiment by dissolving the proper amount of powder immediately into full Eagles’s minimum essential medium supplemented with 20% fetal bovine serum (FBS). The DSS-containing media was mixed thoroughly until fully dissolved using Whatman UNIFO 25 sterile syringe filters (PVDF membrane, 0.22 µm pore size; Cat#WHA9913-2502, Cytia/Whatman, Marlborough, MA, USA) under aseptic conditions. Fully differentiated Caco-2 monolayers cultured in 12-well plates, 8-well chamber slides, or on polyester semipermeable Trans well inserts can be used to induce epithelial damage. Following complete differentiation, the existing culture media were completely removed and replaced with freshly prepared DSS-containing media at the designated concentrations. To identify the dose and exposure duration needed to cause moderate epithelial damage without causing excessive cytotoxicity, preliminary optimization experiments were performed. During preliminary optimization, lower intermediate DSS concentrations (1.0% and 1.5% [*w*/*v*]) were also evaluated but did not produce sufficient and consistent barrier disruption under the experimental conditions. Based on these preliminary observations, together with concentrations reported in existing Caco-2 DSS models. Based on the results, 2.5%(*w*/*v*) DSS for 24 h was selected for all downstream experiments. Cells were incubated with DSS-containing media at 37 °C in a humidified incubator with 5% CO_2_. Following DSS treatment, cells were processed directly for downstream studies such as TEER measurements, LDH cytotoxicity assay, Western blot, immunocytochemistry, and reactive oxygen species (ROS) assessment.

### 4.3. Recombinant IL-6 Treatment

To assess the role of IL-6 in epithelial barrier restoration during continued DSS exposure, differentiated Caco-2 monolayers were treated with recombinant human IL-6 (Bio-Techne, Minneapolis, MN, USA) at the designated concentrations. For rIL-6 treatment experiments, cells were first subjected to 2.5%(*w*/*v*) DSS for 24 h to cause epithelial damage. Following DSS exposure, rIL-6 was directly added to the existing DSS-containing media to achieve the final treatment concentrations. IL-6 was used at final doses of 1 ng/mL and 5 ng/mL in line with the experimental design. The rIL-6 concentrations (1 and 5 ng/mL) were selected based on our previous in vitro study, in which Caco-2 cells were treated with a range of rIL-6 concentrations (0.16–10 ng/mL) and their effects on cellular apoptosis were evaluated as experimental treatment conditions and were not intended to represent physiological circulating IL-6 concentrations in humans [44]. In the present study, 1 and 5 ng/mL were used as lower and higher experimental concentrations, respectively, to evaluate concentration-dependent effects on epithelial barrier function. After rIL-6 addition, cells were incubated for the specified treatment duration at 37 °C with 5% CO_2_ under standard culture conditions. Untreated differentiated Caco-2 monolayers and DSS-treated cells with or without rIL-6 were used as experimental controls and treatment groups, respectively. Cells and supernatants were obtained at the end of treatment for further examinations, such as Western blot, immunochemistry, and oxidative stress evaluation.

### 4.4. Butyrate Treatment

Butyrate sodium salt (Cat# 303410-5G, Sigma-Aldrich, St. Louis, MO, USA) was used for differentiated Caco-2 monolayers to evaluate its impact on epithelial responses in Caco-2 monolayers during continued DSS exposure. Fresh butyrate was prepared by dissolving the compound in sterile phosphate-buffered saline (PBS). For treatment studies, Caco-2 cells were exposed to 2.5%(*w*/*v*) DSS for 24 h. After that, 1 mM and 10 mM Butyrate were directly added to DSS-containing media without removing them to evaluate their effects on epithelial barrier restoration during continued DSS exposure. Butyrate concentrations (1 mM and 10 mM) were chosen based on prior work conducted in our lab using Caco-2 cells, where these values were assessed for their effects on tight junction regulation, oxidative stress, and epithelial barrier integrity [19]. These earlier results led to the selection of 1 mM and 10 mM as the lower and higher experimental concentrations, respectively, for assessment in the current DSS-induced epithelial damage model. Here, 10 mM is within the millimolar range of butyrate concentrations recorded in the human intestinal lumen, despite the fact that these doses were chosen based on our prior in vitro research rather than to precisely replicate physiological exposure [55]. Cells were subsequently incubated at 37 °C with 5% CO_2_. Again, untreated cells and DSS-treated cells without butyrate treatment were used as controls. After the treatment, cells were processed for downstream molecular assays.

### 4.5. Cell Permeability Assay

Transepithelial electrical epithelial resistance (TEER) was used to determine cell permeability following treatment with rIL-6 and butyrate in DSS-treated Caco-2 cell monolayers. For TEER experiments, cells were seeded in trans well inserts 0.3 µm pore size; Cat#662630, Griener Bio-One, Monroe, NC, USA), which were placed in normal 12-well plates. They were allowed to fully differentiate for 14–21 days, with media being changed every other day until a stable epithelial monolayer was formed. The apical compartment received treatment media, while the basal compartment received fresh complete media to maintain cell viability. Following the experimental treatment period, TEER measurements were recorded using an epithelial Volt-ohmmeter, Millicell ERS-2 electrical resistance system (MilliporeSigma, Burlington, MA, USA). For each reading, the electrodes were inserted at a right angle, with the shorter tip of the electrode placed in the apical compartment, taking care not to disturb or physically contact the epithelial monolayer, and the longer electrode placed in the basal compartment. The integrity of the epithelial barrier was determined based on the resistance measured across the monolayer, where higher resistance values indicated a more intact epithelial barrier. TEER values were calculated as Ohm(resistance×cm^2^ (surface area of the filter), after the resistance was subtracted by the value of the filter without a cell monolayer. TEER values were used as an indicator of epithelial permeability and barrier function.

### 4.6. Lactate Dehydrogenase Assay

The LDH-Glo^TM^ Cytotoxicity Assay (Promega, Madison, WI, USA) was used to measure cell cytotoxicity after DSS treatment in accordance with the manufacturer’s instructions. Cell culture supernatants were carefully collected from each condition at the end of the treatment period, without disturbing the cells, and used for LDH analysis. To account for background luminescence from LDH in the media, wells containing culture medium devoid of cells were added as negative controls. Then, 50 µL of each diluted sample was added to a 96-well opaque-walled assay plate after collected samples were diluted as needed using LDH storage Buffer. Next, each well received an equal volume (50 µL) of LDH Detection reagent. The assay plate was incubated for 60 min at room temperature, and luminescence was detected using a cell culture microplate reader. Following DSS exposure, the amount of LDH released into the supernatant served as an indicator for cytotoxicity and cell membrane damage. Where applicable, the following formula was applied to determine percent cytotoxicity:

$$Percent\;Cytotoxicity=100\times \frac{\left(Expermental\;LDH\;Release-Medium\;Background\right)}{\left(Maximum\;LDH\;Release\;Control-Medium\;Background\right)}$$

### 4.7. Western Blot Analysis

Western blot analysis was used to assess NADPH oxidase 1 (NOX1) protein expression. Cells were lysed using Cell Lysis Buffer (Cat# 9803, Cell Signaling Technology, Danvers, MA, USA) supplemented with protease inhibitors after being washed with cold PBS at the end of the treatment period. Cell lysates were collected, transferred to microcentrifuge tubes, and stored at −80 °C until further analysis. The Thermo ScientificTM PierceTM BCA Protein Assay Kit (Thermo Fisher Scientific, Waltham, MA, USA) according to the manufacturer’s instructions. Equal amounts of total protein were mixed with loading buffer, denatured by heating, and separated by SDS-PAGE on polyacrylamide gels. Next, proteins were transferred onto polyvinylidene difluoride (PVDF). After transfer, membranes were blocked using EveryBlot Blocking Buffer (Bio-Rad Laboratories, Hercules, CA, USA) and incubated with a primary antibody against NOX1 overnight at 4 °C. Following washing, membranes were incubated with an HRP-conjugated secondary antibody. For loading control detection, membranes were also incubated with HRP-conjugated GAPDH antibody (Bio-Rad Laboratories, Hercules, CA, USA). An enhanced chemiluminescence (ECL) detection system was used to visualize protein bands, and images were acquired using a gel documentation system. Band intensities were quantified using ImageJ software for MacOS (1.53k), and NOX1 expression was normalized.

### 4.8. Quantitative Real-Time PCR (qRT-PCR) Analysis

qRT-PCR was performed to analyze gene expression. Total RNA was extracted using TRIzol reagent (Cat# 15596026, Thermo Fisher Scientific, Waltham, MA, USA) according to the manufacturer’s protocol with minor modifications. In simple terms, cold TRIzol was used to lyse the cells, chloroform (Cat# C607-4, Fisher Scientific, Fair Lawn, NJ, USA) was used for phase separation, and ice-cold isopropyl alcohol (Cat# A461-4, Fisher Scientific, Waltham, MA, USA) was used for RNA precipitation. RNase-free (DEPC-treated) water (Cat# AM9906, Thermo Fisher Scientific, Waltham, MA, USA) was used to resuspend the RNA pellet after it had been cleaned with 70% ethanol and allowed to air-dry. RNA concentration and purity were determined using a NanoDrop OneC Spectrophotometer (Thermo Fisher Scientific, Waltham, MA, USA). RNA samples were further purified by LiCl precipitation using InvitrogenTM LiCl Precipitation Solution (7.5 M) (Thermo Fisher Scientific, Waltham, MA, USA) to increase RNA purity and reduce possible DSS interference. Briefly, the RNA sample was well mixed with 0.1 volume of 3 M sodium acetate (pH 5.2) and 2 volumes of pre-chilled 100% ethanol after LiCl purification. It was then incubated at −20 °C for 30 min and centrifuged at 14,000× *g* for 30 min at 4 °C. The pellet underwent washing with 70% ethanol, was air-dried, and subsequently resuspended in RNase-free water. The A260/A280 ratio was used to evaluate the quality of the RNA; values above 1.8 were deemed acceptable. Using the High-Capacity cDNA Reverse Transcription Kit (Cat# 4368814, Applied Biosystems, Waltham, MA, USA), complementary DNA (cDNA) was synthesized from 1000 ng of pure RNA in accordance with the manufacturer’s instructions. The resulting cDNA was diluted using nuclease-free water.

PowerUpTM SYBR Green Master Mix (Cat# A25742, Applied Biosystems, Waltham, MA, USA) was used for quantitative PCR using a QuantStudioTM 3 Real-Time PCR System (Applied Biosystems, Waltham, MA, USA). For gene expression analysis, primers obtained from Bio-Rad (Hercules, CA, USA) were utilized. The 2^−Δ∆Ct^ method, with GAPDH used as the internal control.

### 4.9. Immunocytochemistry Analysis

Immunocytochemistry was used to assess intracellular reactive oxygen species (ROS) levels in treated Caco-2 cells, as well as the expression and localization of NOX-1 and Claudin-2. Cells were seeded at a density of (1 × 10)^4^ cells per chamber onto 8-well chamber slides (Cat# 354108, Corning, Corning, NY, USA) and allowed to differentiate for 14 days. During continued DSS exposure, cells were subjected to the designated treatment conditions, including 1 mM and 10 mM butyrate (BA) and 1 ng/mL and 5 ng/mL recombinant IL-6 (rIL-6), as described in previous sections. DSS remained present in the culture medium during these subsequent treatments; therefore, these experiments evaluated the effects of BA and rIL-6 on epithelial cells during continued DSS exposure rather than following DSS withdrawal. Cells were fixed with 10% buffered formalin (Cat# 245-684) for 10 min at the end of the treatment period, and then they were rinsed three times with phosphate-buffered saline (PBS). After being permeabilized with 0.2% Triton X-100 (Cat# 85111), the cells were once more washed with PBS. Also, 10% goat serum (Cat# 50062Z, Invitrogen, Thermo Fisher Scientific, Waltham, MA, USA) was used to prevent non-specific binding. Cells were incubated with primary antibodies against Claudin-2 (Cat# 710221, Invitrogen, Thermo Fisher Scientific, Waltham, MA, USA) and NOX1 (Cat# PA5-103220, Thermo Fisher Scientific, Waltham, MA, USA). Cells were washed with PBS and incubated with FITC-conjugated goat anti-rabbit IgG secondary antibody (Cat# 65-6111, Invitrogen, Thermo Fisher Scientific, Waltham, MA, USA) following primary antibody incubation. After washing the slides multiple times with PBS, slides were mounted using VECTASHIELD Antifade Mounting Medium with 4’,6-diamidino-2-phenlindole (DAPI) (Cat# H-1200-10, Vector Laboratories, Newark, CA, USA), and coverslips were applied. An AMScope IN480 series inverted epifluorescence microscope (AmScope, Irvine, CA, USA) was used to take fluorescent pictures. NIH ImageJ software was used for image processing and fluorescence channel merging.

For the detection of intracellular ROS, DHE staining was performed. Following fixation, permeabilization, and washing steps as described above, cells were incubated with 1 µM DHE (Cat# D23107, Thermo Fisher Scientific, Waltham, MA, USA) for 25 min. Cells were then washed three times with PBS, mounted with VECTASHIELD mounting medium, and imaged using the same fluorescence microscope. Increased red fluorescence intensity was interpreted as elevated ROS production.

### 4.10. Enzyme-Linked Immunosorbent Assay (ELISA)

A high-sensitivity ELISA kit (Thermo Fisher Scientific, Waltham, MA, USA) was used to assess the amount of interleukin-6 (IL-6) in cell culture supernatants in accordance with the manufacturer’s instructions. After DSS treatment, supernatants were extracted from Caco-2 cells at specified intervals. A Multiskan^TM^ FC microplate reader (Thermo Fisher Scientific, Waltham, MA, USA) was used to quantify absorbance at 450 nm after samples were processed according to the ELISA protocol. IL-6 concentrations were determined based on standard curves using the standards provided in the kit. All experimental conditions were performed in technical replicates (n = 4).

### 4.11. Statistical Analysis

All experiments were performed with at least three independent biological replicates, unless otherwise noted. Data are reported as mean ± standard deviation (SD). Statistical analyses were performed using GraphPad Prism software version 10.1.2 (GraphPad Software, Boston, MA, USA). Comparisons across various groups were performed using one-way analysis of variance (ANOVA) followed by Bonferroni adjustment for multiple comparisons. For comparisons between two groups, an unpaired two-tailed Student’s *t*-test was used where appropriate. A *p*-value < 0.05 (*), *p*-value < 0.01 (**), *p*-value < 0.001 (**) and *p*-value < 0.0001 (****) was considered statistically significant.

## Data Availability

The raw data supporting the conclusions of this article will be made available by the authors on request.

## Abbreviations

The following abbreviations are used in this manuscript:

ANOVA	Analysis of variance
BA	Butyric Acid
CD	Crohn’s Disease
CLDN2	Claudin-2
DAPI	4′,6-diamidino-2-phenylindole
DSS	Dextran Sodium Sulfate
GAPDH	Glyceraldehyde-3-Phosphate Dehydrogenase
IL-6	Interleukin-6
rIL-6	Recombinant Interleukin-6
MAP	*Mycobacterium avium paratuberculosis*
NOX1	NADPH oxidase 1
ROS	Reactive Oxygen Species
STAT	Signal Transducer and Activator of Transcription
TEER	Transepithelial electrical resistance
UC	Ulcerative colitis
ZO-1	Zonula Occludens-1
